# Widespread introgression in Chinese indigenous chicken breeds from commercial broiler

**DOI:** 10.1111/eva.12742

**Published:** 2019-01-22

**Authors:** Chunyuan Zhang, Deng Lin, Yuzhe Wang, Dezhi Peng, Huifang Li, Jing Fei, Kuanwei Chen, Ning Yang, Xiaoxiang Hu, Yiqiang Zhao, Ning Li

**Affiliations:** ^1^ Beijing Advanced Innovation Center for Food Nutrition and Human Health China Agricultural University Beijing China; ^2^ State Key Laboratory for Agrobiotechnology, College of Biological Sciences China Agricultural University Beijing China; ^3^ Institute of Poultry Science Chinese Academy of Agricultural Sciences Yangzhou China; ^4^ National Engineering Laboratory for Animal Breeding China Agricultural University Beijing China

**Keywords:** Chinese indigenous chickens, haplotype similarity, Introgression

## Abstract

Chinese indigenous chickens (CICs) constitute world‐renowned genetic resources due to their excellent traits, including early puberty, good meat quality and strong resistance to disease. Unfortunately, the introduction of a large number of commercial chickens in the past two decades has had an adverse effect on CICs. Using the chicken 60 K single nucleotide polymorphism chip, we assessed the genetic diversity and population structure of 1,187 chickens, representing eight Chinese indigenous chicken breeds, two hybrid chicken breeds, two ancestral chicken breeds, two commercial populations and additional red jungle fowl. By investigating haplotype similarity, we found extensive gene introgression from commercial broiler to almost all CICs. Approximately 15% of the genome, on average, of CICs was introgressed, ranging from 0.64% for Tibetan chicken to 21.52% for Huiyang Bearded chicken. Further analysis revealed signals consistent with positive selection in the introgression loci. For the first time, we systematically mapped and quantified introgression from commercial broiler to CICs at the whole genome level. Our data provided a usable resource for chicken genetic diversity, and our findings indicated a dire need for protecting the genetic resources of CICs.

## 1 INTRODUCTION

Genetic exchange occurs frequently in nature. Since introgression will introduce new genetic material, it increases genomic diversity. In the case in which the donor allele is adaptive, it will also augment the fitness of the receptor, which is referred to as *adaptive introgression*. It is well known that the genome of modern humans contains genetic introgression from Neanderthal and Denisovan hominins, which is beneficial to adaptation to the new environment of human ancestors during migration (Huerta‐Sánchez et al., 2014; Sankararaman et al., 2014). Genetic introgression from Denisovans was detected, which assisted them to adapt to the high elevation of the Tibetan plateau (Huertasanchez et al., 2014). In animals, extensive gene introgression has been reported during domestication, either between domestic animals and their wild counterparts (Frantz et al., 2015) or between different domestic breeds (Parker et al., 2017). As an example, the improved litter size of Large White pigs was attributed to the *AHR* haplotype introgressed from Chinese indigenous pigs (Bosse et al., 2014). Human‐mediated introgression is also very common in modern breeding practice, which significantly accelerates the process of breeding and shortens genetic advances. However, from the perspective of protecting genetic resources, introgression might not always be advantageous. For example, several reports identified a loss of genetic diversity or reduced viability of wild animals after introgression from commercial populations, including pigs (Berthouly‐Salazar et al., 2012), wild lake trout (Muhlfeld et al., 2009) and dark honey bee (Muñoz et al., 2015).

It has been widely acknowledged that domestic chickens most likely descended from red jungle fowl (*Gallus gallus*) (Fumihito et al., 1994, 1996), with additional evidence that other jungle fowl species also contributed to chicken domestication (Eriksson et al., 2008). Archaeological evidence has revealed that the domestication of chickens can be traced back to approximately 8,000 years ago (Miao et al., 2013; West & Zhou, 1988), Chinese indigenous chickens (CICs) are renowned genetic resources due to their important traits, including early puberty, good meat quality and strong resistance to disease. However, a major disadvantage of CICs is that their growth rate is significantly inferior to modern commercial broilers, and therefore, they are less competitive in the market. Upgrading the native chicken using high producing commercial populations was regarded as the quickest way of achieving genetic improvement for growth, as well as for increasing egg and meat production (Khan, 2008). With this strategy, new hybrid chickens were successfully created, such as the Shiqi Za chicken, Kuaida Silkie and Three‐Yellow chicken. The hybrid chickens retain most characteristics of indigenous chickens, but grow at a much faster rate (Ahmed, Saleem, & Zahid, 2012). Unfortunately, not all hybridizations were well organized. For the past decade, the problem of unexpected hybridization became especially severe in rural regions, where organized breeding practices were generally absent. In fact, analysis on microsatellites revealed that China's chicken resource structure changed after the introduction of a large number of foreign commercial chickens in the 1980s. According to this report, Huiyang Bearded chicken suffered from admixture and decreasing population size (Huang et al., 2016), and this is a common phenomenon in developing countries (Alqamashoui, Simianer, Kadim, & Weigend, 2014).

Although it was found that CICs might have been threatened by commercial chickens, the extent of “genetic pollution” remains unclear. In this study, we studied genomewide introgression from commercial broiler to China indigenous chickens using the 60 K chicken chip for 1,187 chickens, comprising eight typical CICs, two hybrid chicken breeds, two ancestral chicken breeds, two commercial populations including one popular broiler and one popular layer, and additional red jungle fowl. By utilizing the haplotype similarity approach, we identified introgression genomic loci into CICs from commercial broiler. Almost all of the CICs are introgressed from commercial broiler to varying degrees. Our results suggest that Chinese indigenous chicken genetic resources are seriously threatened. For this reason, there is an urgent need to develop systematic conservation and utilization strategies to protect Chinese indigenous chicken genetic resources.

## 2 MATERIALS AND METHODS

### 2.1 Ethical statement

Blood samples from chickens were collected from the brachial vein on living birds, following a standard venipuncture protocol approved by the Animal Welfare Committee of China Agricultural University (permit number: XK622). All of the chickens used in the current study were fed and handled according to the guidelines for the care and use of experimental animals established by the Ministry of Science and Technology of the People's Republic of China (approval number: 2006‐398).

### 2.2 Sample collection

**Figure 1:**
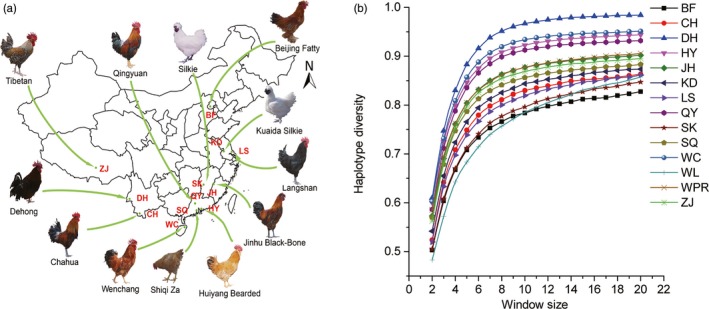
Samples and haplotype diversity of CICs. (a) Geographic location of CICs used in our study; (b) haplotype diversity in varying window size. The order from top to bottom: DH, WC, HY, QY, WPR, JH, ZJ, SQ, KD, CH, LS, SK, BF and WL

The diversity panel comprised 10 representative CICs, two ancestral chicken breeds (Table 1; Figure 1a; Supporting Information Table S1) and two commercial populations (Table 1; Supporting Information Table S1). Dehong (DH) and Chahua (CH) are ancestral chicken breeds in human settlement areas originally from Yunnan province. White Plymouth Rock (WPR) and White Leghorn (WL) are commercial populations, and all others are CICs. Among CICs, Shiqi Za (SQ) and Kuaida Silkie (KD) were hybridized from Shiqi (not included in our sample) and Silkie (SK) with WPR, respectively, while both retain most phenotypic characteristics of CICs. All samples, with the exception of WL, were collected from the National Chicken Genetic Resources Center in Jiangsu Province, China. The raw genotype data for WL were obtained from Liu et al., (2011). Red jungle fowl was provided by the Animal Breeding and Genomics Centre, Wageningen University and Research Centre, Netherlands. Due to the small simple size (*n* = 4), it was only used for population analysis. In total, 1,747 samples were included (Supporting Information Table S1).

**Table 1:** Summary of samples and genetic diversities

Breed	Acronym	Sample size	Province	*P_n_* (%)	ROH (Mb)
Chahua	CH	88	Yunnan	78.51	4.51
Dehong	DH	46	Yunnan	92.04	4.99
Huiyang Bearded	HY	83	Guangdong	92.30	4.32
Jinhu Black‐Bone	JH	74	Fujian	87.28	4.83
Beijing Fatty	BF	79	Beijing	75.35	4.44
Langshan	LS	89	Jiangsu	78.22	4.30
Qingyuan	QY	90	Guangdong	88.63	5.23
Silkie	SK	75	Jiangxi	73.36	4.68
Tibetan	ZJ	71	Tibetan	83.34	4.69
Wenchang	WC	95	Hainan	93.05	5.67
Shiqi Za	SQ	93	Guangdong (hybridization)	87.21	5.16
Kuaida Silkie	KD	66	Jiangsu (hybridization)	82.72	4.93
White Leghorn	WL	54	Commercial laying hens	67.39	3.85
White Plymouth Rock	WPR	180	Commercial broilers	91.54	4.87
Red Jungle Fowl	GG	4	*Gallus gallus*	—	—
Total		1,187			

### 2.3 Genotyping and quality control

Genomic DNA was extracted from the blood samples using a standard phenol–chloroform extraction protocol. Our DNA samples were genotyped by Landmarks Inc., Quebec, Canada, using Illumina chicken 60 K single nucleotide polymorphism (SNP) BeadChips (57,636 SNPs; Groenen et al., 2011). Quality control for the genotype data was performed using the Genotyping Module of Illumina GenomeStudio (v2011.1). To obtain more qualified data, the clusters for 813 SNPs in our data were manually edited (Supporting Information Figure S1). A series of quality control measures (filters) were then applied. SNPs with a minor allele frequency (MAF) <0.05 or call frequency <95% across all samples or cluster separation value <0.3 were excluded. We set the threshold of heterozygosity cluster intensity as AA R mean >0.2, AB R mean >0.2, BB R mean >0.2. Mitochondrial SNPs, W chromosome SNPs and Z chromosome SNPs were excluded. Finally, 36 samples with a call rate <95% were excluded (Supporting Information Figures S2–S4, Supporting Information Table S1). All SNPs in the chip were mapped back to Gallus_gallus‐5.0/galGal5 (December 2015) assembly to assign genome coordinates. Duplicated SNPs (Supporting Information Table S2) or SNPs that no longer existed in the dbSNP database (Supporting Information Table S3) were excluded. We also removed 11 samples as outliers that were three standard deviations away from the breed mean on any of the first two principal components, by projecting all individuals onto principal component space using SMARTPCA (Supporting Information Figure S5, Supporting Information Table S1). The SNPs were pruned using a threshold of pair‐wise *r* ^2^ = 0.2 within 50 SNP sliding windows and were shifted every five SNPs across the genome with PLINK (v1.9) (Chang et al., 2015; Gaunt, Rodríguez, & Day, 2007; Purcell et al., 2007). Pairs of closely related individuals (proportion IBD (PI_HAT) >0.5) were carefully checked, and 513 samples were excluded due to closeness and lower call rate (Supporting Information Table S1) using the pruned data. Finally, 46,239 informative SNPs for the 1,187 samples (Table 1; Supporting Information Table S1) were retained for further analyses. The distribution of the raw/qualified SNPs on the chicken physical map is shown in Supporting Information Table S4.

### 2.4 Haplotype inference

Phasing (Browning & Browning, 2007) and imputation (Browning & Browning, 2016) of missing genotypes were conducted by BEAGLE (v4.1) for the whole population. For the phased genome, we broke the whole genome into windows without overlapping, and we counted the number of distinct haplotypes within each window for each population. Considering that the choice of window sizes might affect results, we attempted different window sizes varying from 2 to 20 SNPs. If the last window of each chromosome contained less than the window size, it was dropped. All analyses based on haplotype were carried out for different window sizes independently.

### 2.5 Genetic diversity indices

Loci with MAF greater than 0.05 within populations were treated as polymorphic. The proportion of polymorphic SNPs (*P_n_*) was calculated using VCFTOOLS (v0.1.12b) (Danecek et al., 2011). Runs of homozygosity (ROH) of each population were computed across the genome for windows with at least 50 SNPs or a minimum length of 500 kb using PLINK (v.1.9) (Purcell et al., 2007), allowing five missing calls and one heterozygous SNP. Haplotype diversity (*H*) was calculated for each population as described by Nei & Tajima, (1981):

$$H=\frac{N}{N\;\text{‐}\;1}\left(1\;\text{‐}\;\sum_{i}x_{i}^{2}\right)$$

where *x_i_* is the haplotype frequency within populations; and *N* is the sample size.

### 2.6 Population analysis

We inferred a population‐level phylogeny using the neighbour‐joining method implemented in the PHYLIP package (v3.696) with 1,000 bootstrap replicates (Felsenstein, 1989). Red jungle fowl was selected as an outgroup in each run. IBS distance was also used to construct the neighbour‐joining tree at the individual level. FIGTREE (Rambaut, 2016) was used to visualize the built tree (v1.4.3). Principal component analysis (PCA) for all chickens was performed using SMARTPCA (Patterson, Price, & Reich, 2006). The pair‐wise population differentiation (Weir and Cockerham's *F*st (Weir & Cockerham, 1984)) between populations was calculated using VCFTOOLS (v3.0) (Danecek et al., 2011) in sliding windows along the genome (100 kb per window, shifted 50 kb per step). Pair‐wise *F*st values between any two populations are shown in Supporting Information Figure S6. Admixture proportions were estimated using a maximum likelihood approach, as implemented in the program ADMIXTURE (v 1.23) (Alexander, Novembre, & Lange, 2009). We ran the analyses by varying genetic clusters from *K* = 2 to *K* = 5, and the outputs were further visualized using GENESIS (Buchmann & Hazelhurst, 2014). The number of markers needed to resolve populations by ADMIXTURE is inversely proportional to the genetic distance (*F*st) between populations. In this study, we have sufficient markers for ADMIXTURE analysis since the authors recommend that 10,000 markers will suffice for populations with *F*st greater than 0.05 (Supporting Information Figure S6).

### 2.7 Genomic IBD sharing

For a preliminary detection of introgression from commercial broiler, we calculated the identity by descent (IBD) sharing using BEAGLE (v4.1) (Browning & Browning, 2013) between CICs and WPR on the whole genome. The genetic distance was converted to physical distance as 300 k/cM in the genome.(Sheng et al., 2013). Only segments with LOD greater than 3 and length greater than 1 cM were kept. Shared IBD were summed up for each pair of CIC and WPR, and further adjusted by the product of the population size of the pair.

### 2.8 Introgression detection from commercial broiler to Chinese indigenous chickens

In order to detect the introgression, we proposed a hypothesis that, if extensive introgression occurred from commercial broiler to CICs, we would observe genomic loci of CICs exhibiting higher haplotype similarity to commercial broiler than to their ancestor populations. Haplotype similarity was evaluated by cumulative chi‐square statistics (Pearson, 1900) between CH versus CICs and CICs versus WPR:

$$\chi^{2}=\sum_{i=1}^{n}\frac{{\left(A_{i}-T_{i}\right)}^{2}}{T_{i}}$$

where *A_i_* and *T_i_* are observed and expected haplotype counts for cell *i*, respectively. Overall, the higher the similarity between two populations, the lower the chi‐square values. We then constructed Δχ^2^ as chi‐square between CH and CICs minus chi‐square between CICs and WPR:

$$\Delta \chi^{2}=\chi_{\text{CHvsCICs}}^{2}\;\text{‐}\;\chi_{\text{CICsvsWPR}}^{2}$$

As the ancestor of Chinese domestic chicken, CICs should share more similarity with CH than with WPR in most genomic regions. So, Δχ^2^ would be less than zero in most genome regions. When introgression occurred from WPR to CICs, in which we were interested in this study, we would observe a Δχ^2^ greater than zero. A permutation test was used to determine the statistical significance of the difference. In detail, we randomly assigned group labels, where sample size in each breed was held constant, and re‐calculated Δχ^2^ statistics for 1,000 times. Based on the null hypothesis of Δχ^2^ less than zero, an empirical *p* value was estimated as *p* = (*n* + 1)/1,001, where *n* was the count of the permutated test Δχ^2^ value greater than the observed Δχ^2^ value. Windows with *p* value lower than 0.001 and Δχ^2^ greater than zero would be regarded as candidate introgressive loci from WPR. Among the candidate regions, we further excluded loci that were overlapped with the loci reported in DH for possible false positives due to common ancestry. The distribution of Δχ^2^ is shown in Supporting Information Figure S7. All of the statistics in the introgression detection analyses were calculated using in‐house scripts. D‐statistic, which was originally developed on site counts to test hybridization between humans and Neanderthals, was shown to be robust to detect gene flow between closely related species (Zheng & Janke, 2018). We also used D‐statistics for the ordered set (CH, CICs, WPR, GG). In this setting, a negative *Z*‐score suggests that gene flow occurred either between CICs and WPR or CH and GG. Results are provided in Supporting Information Table S5.

### 2.9 Randomness test for candidate introgression loci

In order to determine whether the introgression loci are randomly distributed or clustered physically in the genome, we used the runs test (Bradley, 1968). The runs test is a nonparametric statistical test that determines whether a data set is from a random process. The formula is as follows:

$$\overset{¯}{R}=\frac{2n_{1}n_{2}}{n_{1}+n_{2}}+1$$

$$S_{R}^{2}=\frac{2n_{1}n_{2}\left(2n_{1}n_{2}-n_{1}-n_{2}\right)}{{\left(n_{1}+n_{2}\right)}^{2}\left(n_{1}+n_{2}-1\right)}$$

$$Z=\frac{R-\overset{¯}{R}}{S_{R}}$$

where *n*_1_ and *n*_2_ are the number of introgressed and nonintrogressed windows, respectively; *R* is the observed number of runs; and $\overset{¯}{R}$ is the expected number of runs. We defined a continuous candidate or noncandidate introgression region as a run. *S_R_* is the standard deviation of the number of runs. For a large‐sample runs test, where *n*_1_ and *n*_2_ are greater than 10, as in our study, the test statistic is compared to a normal distribution. So, if the absolute value of *Z* is greater than 1.96, the null hypothesis of randomness is rejected with *p* < 0.05 (2.58 for *p* < 0.01). The test was run for each population independently.

### 2.10 Positive selection detection

In order to test for positive selection signals, we employed the XPEHH test, which is powerful in detecting ongoing selective sweeps (Voight, Kudaravalli, Wen, & Pritchard, 2006). The unstandardized XPEHH statistic was calculated between CH and CICs as:

$$\text{unstandarized}\;\text{XPEHH}=\ln\left(\frac{{\text{iHH}}_{A}}{{\text{iHH}}_{B}}\right)$$

where iHH_A_ and iHH_B_ are integrated EHH of a given core SNP in population A (CH hereof) and B (one of the CICs hereof), respectively. A positive value of XPEHH suggests selection in population A, while a negative value for selection in population B. We use the software XPEHH, which was developed by Pickrell et al. (2009) to estimate unstandardized XPEHH statistics. Since our data were not as dense as the data used by Sabeti, we used the “‐nd” option to disable the correction factor according to the author's recommendation. Unstandardized XPEHH statistics was *Z*‐score transformed, and p values of SNPs were estimated directly from the normalized distribution. We used the same window as described in the introgression detecting analysis. Windows with three or more significant core SNPs (*p* value <0.05) were classified as candidate positive selection regions.

### 2.11 Simulations

To evaluate the performance of introgression detection using the haplotype similarity approach, we performed simulations using forward simulation software SLiM (v2) (Haller & Messer, 2017). We considered panmictic populations of diploid individuals with genome length 1 × 10^6^ bp. The mutation rate was set to 1 × 10^−8^ per base pair per generation, and the recombination rate was set to 3 × 10^−7^ per base per generation. We simulated chromosomes with the following demographic model (Supporting Information Figure S8): (a) an ancestral population P1 with a size of 5,000 was burned in for 50,000 generations; (b) 2,000 individuals of population P2 split from the ancestral population P1 without migration until 100,000 generations; (c) the P2 admix with P1 during recent 200 generations at varying migration rate from 0.001 to 0.009 with a step of 0.001 and (d) sample 100 diploids in each population at 100,200 generations. Three scenarios were simulated with different positive selection coefficients (s = 0, s = 0.001 and s = 0.002). Benefit SNPs account for 0.1% of the genome. Each simulation runs for 100 times. Since SLiM allowed tracking of all new mutations during simulation, introgression can be assessed by calculating the P2 original SNPs frequency. We applied both allele frequency weighted and haplotype similarity methods to calculate the introgression ratio from P2.

## 3 RESULTS

### 3.1 Genetic diversity of Chinese indigenous chickens

We selected 656 representative CICs from eight populations, 159 hybrid chickens from SQ and KD, 134 wild chickens from CH and DH, 234 commercial chickens from WPR and WL, and an additional red jungle fowl (*n* = 4) (Figure 1a; Table 1; Supporting Information Table S1). Three genetic diversity indices were calculated to evaluate the intra‐population diversity (Table 1): the proportion of polymorphic loci (*P_n_*), ROH and haplotype diversity (*H*). Populations with the highest *P_n_* were WC, HY, DH and WPR, which were all above 90%. WL, SK and BF exhibited the lowest values. The *P_n_* value of LS and CH was higher than other CICs, such as QY, JH, SQ, ZJ and KD. ROH length distribution showed different patterns, with the average longest for WC (5.67 Mb) and the shortest for WL, which indicated complex breeding history of different chickens (Supporting Information Figure S9). As shown in Figure 1b, DH had the highest haplotype diversity, and WL had the lowest. *H* increased rapidly with the increase of window size, until the window size reached 10 SNPs, of which the *H* tended to be stable. However, the order of *H* in different populations tended to be unchanged under various window sizes. We only performed haplotype similarity in subsequent analysis on varying window size from 2 to 10 SNPs. Overall, results from *P_n_* and *H* were in good agreement with each other. It is worth noting that the genetic diversity of CICs was much higher than that of commercial chickens in previous studies (Ponsuksill, Wimmers, Schmoll, Horst, & Schellander, 1999; Vanhala, Tuiskula‐Haavisto, Elo, Vilkki, & Maki‐Tanila, 1998). However, for the samples that we collected in this study, which are approximately 20 years later, we observed decreased genetic diversities for CICs. In addition, some commercial chicken, such as WPR, exhibited even higher diversity compared to CICs.

### 3.2 Evidence of introgression from commercial broiler

**Figure 2:**
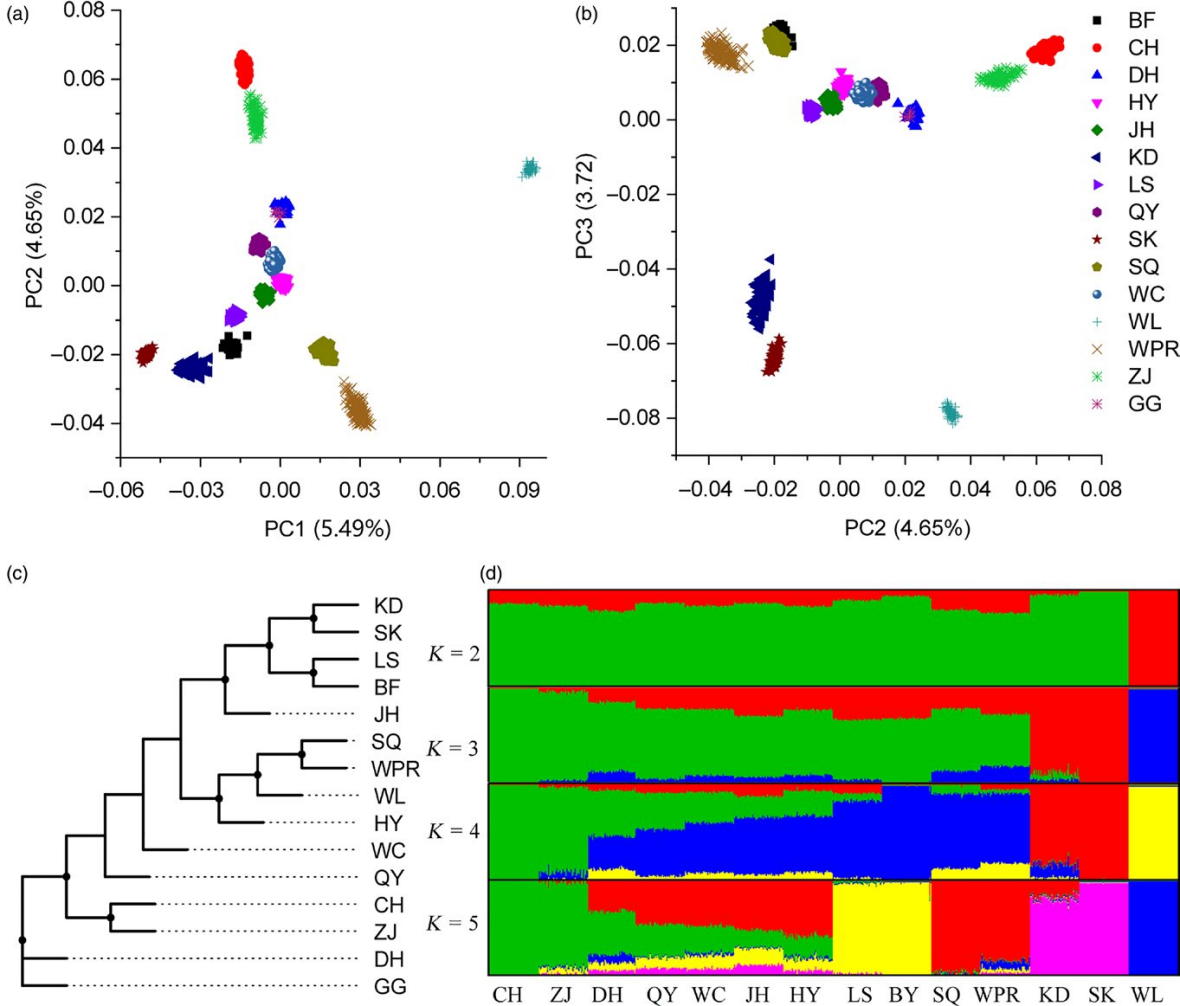
Population structure for all chickens. (a) Principal component analysis (PCA) plot based on PC1 against PC2 and (b) PC2 against PC3; (c) phylogenetic relationships for all populations; (d) genetic structure and individual ancestry. Colours in each column represent ancestry proportion over the range of population sizes *K* = 2–5

Principal components analysis (PCA) on genotypes revealed that WL was evidently separated from CICs in the first principal component (Figure 2a). SK differed from other CICs, which is coincident with its unique history. As a hybrid breed from SK and WPR, the projection of KD samples was located between SK and WPR in the PC plot, consistent with our expectation (Figure 2a,b). However, an unanticipated closeness was observed between certain CICs and WPR in PCA. In agreement with the result from PCA, the neighbour‐joining tree showed that CICs from the south or north were well separated, except HY and SQ, which clustered with commercial chickens (Figure 2c; Supporting Information Figure S10). In a separate population structure analysis, WL first separated from all other chickens. From *K* = 3, WPR exhibited a large shared ancestry proportion with many CICs, and this was the case with increased *K* (Figure 2d; Supporting Information Figure S11). Almost all CICs obtained a negative *Z*‐score in D‐statistics, except DH (Supporting Information Table S5), which provided some information about gene flow between CICs and WPR. Since there is no record of genetic contribution from CICs to WPR, these results suggest the possibility of introgression from WPR into CICs.

IBD refers to identical DNA segments inherited from a common ancestor without recombination and has thus been tentatively utilized to detect introgression both in animals (Bosse et al., 2014) and plants (Ferdy & Austerlitz, 2002). SQ and KD, as recorded crossbreeds with WPR, displayed high IBD sharing with WPR (10.37 and 12.73 Mb, respectively). Other indigenous breeds, such as HY, JH and BF, also exhibited substantial IBD sharing with WPR (Table 2), while CH, DH and ZJ exhibited low IBD sharing with WPR (5, 90 and 1.2 Kb, respectively). WL displayed negligible IBD sharing with all CICs (Supporting Information Figure S12). It is worth noting that IBD reported pairwise haplotype sharing at the individual level. Since we are interested in detecting the signature of extensive and detailed introgression of commercial broiler to CICs at the population level, an alternative approach is preferred.

**Table 2:** Statistics of the candidate introgression regions from WPR

Breed	Mean IBD length with WPR (Mb/ind)	*Z* *	Pearson correlation coefficient	Introgression ratio of whole genome (%)	Introgression ratio of PS regions (%)	PS ratio of whole genome (%)	PS ratio of introgression regions (%)
BF	0.97	−6.97	0.10 (*p* = 0.003)	18.41	15.54	4.98	4.20
HY	0.97	−5.09	0.10 (*p* = 0.002)	21.52	26.90	4.29	5.36
JH	1.12	−3.23	0.13 (*p* < 0.001)	18.32	18.44	4.90	4.93
KD	12.73	−4.37	0.10 (*p* = 0.002)	23.98	23.02	4.82	4.63
LS	0.09	−1.91	0.13 (*p* < 0.001)	17.81	17.03	4.99	4.77
QY	0.03	−3.16	0.11 (*p* < 0.001)	12.58	18.46	4.25	6.23
SK	0.00	−3.85	0.07 (*p* = 0.037)	12.99	14.00	4.98	5.36
SQ	10.37	−8.21	0.26 (*p* < 0.001)	47.92	45.29	4.86	4.59
WC	0.21	−5.82	0.13 (*p* < 0.001)	15.51	14.56	4.04	3.79
ZJ	0.00	−0.79	−0.02 (*p* = 0.618)	0.64	0.66	4.96	5.08

Note

PS: positive selection.

* Absolute value of *Z* larger than 1.96 will be considered as significant *p* value <0.05 (2.58 for very significant *p* value <0.01).

### 3.3 Quantifying introgression using haplotype similarity

To better elucidate the extensive and detailed introgression, we developed a haplotype similarity‐based method. In this method, the whole genome SNPs was split into nonoverlapping windows (varying from 2 to 20 SNPs in different runs). Phasing and distinct haplotypes counting within each window size were performed independently, followed by subsequent chi‐square statistics calculation between CH versus CICs and CICs versus WPR (details in Section  2). The process is shown in Supporting Information Figure S13. The basic concept is that, if extensive introgression occurred from commercial broiler to CICs, we would observe genomic loci of CICs that exhibit higher similarity of haplotype structure to commercial broiler than to their wild ancestors or close wild populations. According to the Chinese Poultry Gazette (Chen, Yang, & Wang, 2011), three breeds, including red jungle fowl, DH and CH, that dwelt in a relatively small area in Yunnan province, were thought to be wild ancestors of CICs. On the other hand, CH, according to (Huo et al., 2014), is genetically closest to red jungle fowl. By checking the haplotype diversity (Figure 1b), estimated ancestry proportion (Figure 2d), IBD sharing (Table 2; Supporting Information Figure S12) and sample availability in this study, CH, which was shown to be least introgressed from commercial broiler, was finally chosen as the wild ancestor for CICs in further analyses. WPR was introduced into China in the late 1980s and later became the most popular commercial broiler in China. Based on the population analyses (Figure 2), as well as the IBD sharing, CICs are very likely introgressed from the WPR, and thus, we next quantified the relationship among CH, CICs and WPR. In most loci, CICs are more closely related to CH, rather than WPR. However, if the loci are of the case in which the introgression occurred from WPR to CICs, the case in which we are most interested, we will observe that CICs are more closely related to WPR than to CH. Simulation data suggest that our method clearly detected recent introgression when the introgression ratio is greater than 5% (Supporting Information Figures S14 and S15).

**Figure 3:**
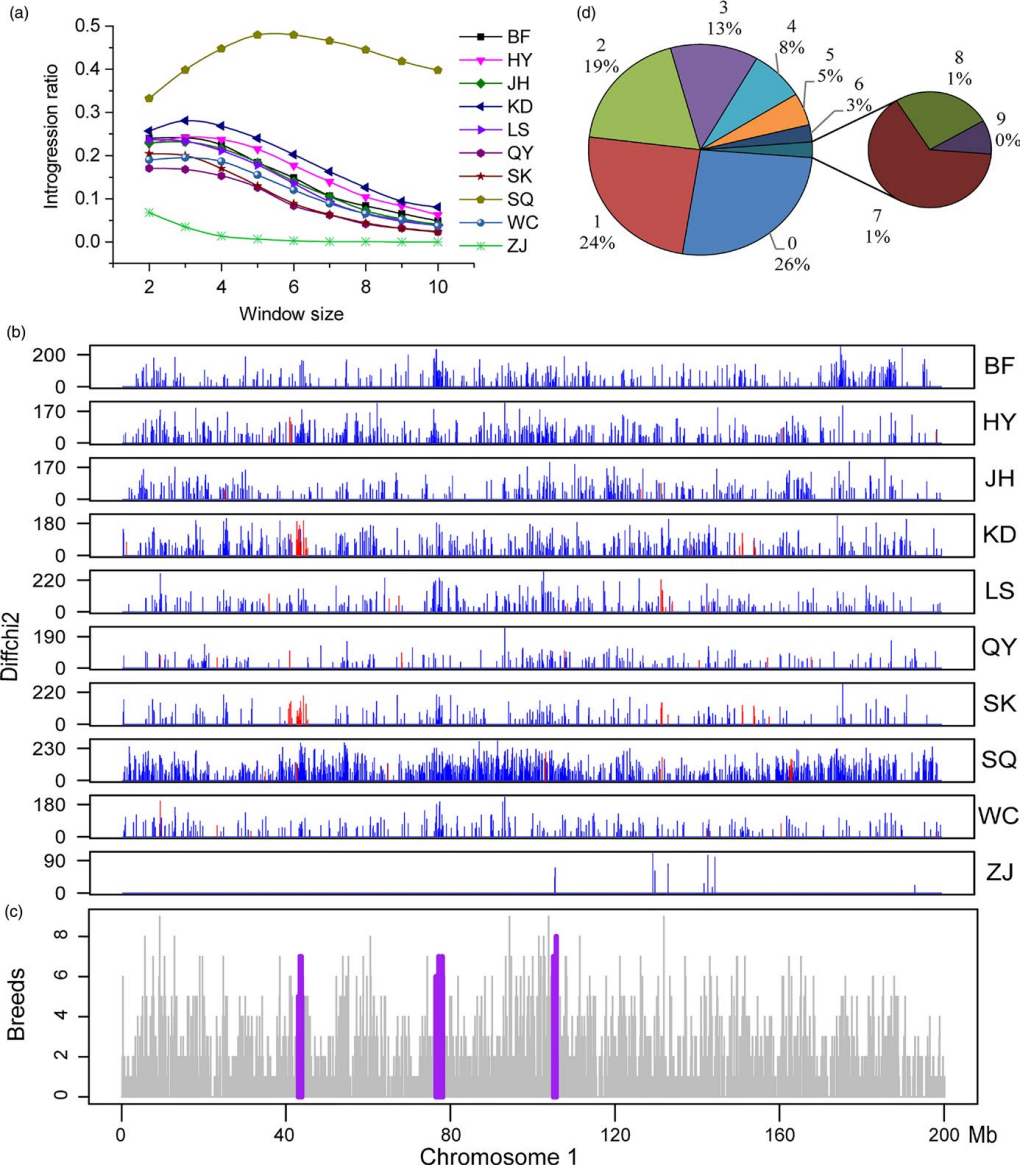
Candidate loci of introgression and positive selection. (a) The statistic of introgression in varying window size for CICs. (b) Physical map and extent of introgression/positive selection on chromosome 1. Each box represents a CIC. The label on the right is the same as the abbreviation in Table 1, and the y‐coordinate is Δχ^2^. The location of introgression is marked with a blue vertical line, and introgression under positive selection is marked with a red vertical line. (c) Introgression frequency in CICs on chromosome 1. Purple regions indicate three long‐range introgression loci which are shared among multiple populations. The first region overlaps with the region under positive selection specifically in KD. (d) Summary of introgression sharing between populations. The number around the pie chart is the population number of introgression sharing and the corresponding ratio

By applying the method described above, we scanned the whole genome for introgression from WPR to each CIC and two hybrid chickens. Permutation tests were performed for each locus to ensure that they constituted true signals. Windows with a *p* value less than 0.001 would be regarded as candidate introgression regions. In order to exclude the possibility of false positives caused by the effect of common ancestor, we removed candidate introgression loci from all CICs if the loci were overlapped with the loci reported in DH (Supporting Information Figure S16). Considering that the choice of window size might affect the results, we repeated the analysis by varying the size from 2 to 10 SNPs. As SQ showed the highest level of introgression when the window size equalled to five SNPs, and the level of introgression decreased in most breeds from five to six SNPs (Figure 3a), we thus determined that the window size of five would be optimal for both sensitivity and specificity, and reported results using the window size of five SNPs, unless otherwise specified.

The final candidate introgression regions from WPR to CICs and hybrid chickens, as well as number of genes overlapping with the introgression regions, are shown in Table 2, Supporting Information Table S6, Figure 3 and Supporting Information Figures S17–S45. WPR mixed with CICs in the IBS tree of these regions further confirmed introgression (Supporting Information Figure S46). 0.64% to 21.52% of the CICs genomes were suggested as being introgressed, with an average of 16.73%. As expected, SQ and KD (47.92% and 23.98%, respectively) were most introgressed, followed by HY, BF, JH, etc. Interestingly, ZJ was least introgressed, with an estimated ratio of 0.64%. On a genome scale, the introgression loci were observed to be shared among populations (Figure 3b,c; Supporting Information Table S6). 9.99% of the introgression loci were shared among at least five CICs, and 0.85% for at least eight CICs, and no region shared by all CICs (Figure 3d; Supporting Information Table S7). Introgression regions that were highly shared among multiple CICs were located on chromosome 1 and chromosome 2 (Figure 3c; Supporting Information Table S8).

### 3.4 Positive selection of introgression loci

For the hundreds of genomic loci that were identified as introgressed, they constituted islands of introgression in each chromosome. We firstly carried out a runs test for randomness to test whether the introgression was random. Results showed that, except for LS (*Z* value = −1.91) and ZJ (*Z* value = −0.79), all other CICs violated the expectation of randomness (Table 2). SQ has the lowest *Z* value of −8.21, and KD had a *Z* value of −4.37, which are far below the threshold of statistical significance, indicating the existence of genomic introgression “hot spots”. Since genes or functional elements tend to cluster in the genome, this would constitute an indication of artificial selection on the introgression loci. These observations were consistent with our expectations because SQ is a hybrid breed that is created purposely, which is also the case for KD. To further determine whether the introgression loci were likely to be associated with gene loci, we counted the number of introgression loci and gene number in every 1 Mb genomic window. By using the Pearson correlation coefficient to measure relatedness between the counts of introgression loci and coding genes, we identified significant correlations across the genome for CICs, except for ZJ (*p* = 0.618; Table 2). SQ exhibited the highest Pearson correlation coefficient among all populations. XPEHH analysis was also performed to detect recently ongoing positive selection signal in the genome. Only windows containing at least three positive selection core SNPs were considered as positive selection windows. We identified a range from 4.04% (WC) to 4.99% (LS) of genomic regions with strong selective signals in each population for the whole genome, or from 3.79% (WC) to 6.23% (QY) of the candidate introgression regions (Table 2; Figure 3b). However, if we checked the ratio of introgression in positive selection regions, the value was elevated to 19% on average, which is consistent with the hypothesis of positive selection after introgression. Of note, we found selection signals in KD on a 0.93 Mb region chr1: 40,853,097–41,778,500, which is identified as a highly shared introgression region (Figure 3b,c).

## 4 DISCUSSION

Introgression, as a sort of double‐edged sword, introduces new genetic materials, which might help to increase adaptability to the environment (Huertasanchez et al., 2014) or improve specific traits (Bosse et al., 2014). On the other hand, gene introgression will reduce specificity, especially for breeds with distinct characteristics (Todesco et al., 2016).

In countries where a mature system for animal breeding is lacking, naïve approaches were more likely adapted by local farmers to cross indigenous breeds with imported commercial chicken. We believe that this not only occurred in the case of chickens, but also pigs (Berthouly‐Salazar et al., 2012). Unfortunately, although a short‐term effect could be observed, it was nearsighted. This sort of hybridization was not an effective way to improve animal performance in the long‐term, due to the incompatibility of crossbred genotypes with inconsistent production systems (Okeno, Kahi, & Peters, 2013). Such activities would, on the contrary, possibly threaten current genetic resources and result in genetic pollution.

Numerous methods exist for detecting introgression (Racimo, Sankararaman, Nielsen, & Huerta‐Sánchez, 2015). D‐statistics or S* or S*‐like approaches (Browning, Browning, Zhou, Tucci, & Akey, 2018) were developed for detecting introgression from ancient humans to modern human genomes (Green et al., 2010; Tajima, 1989). Because of the long divergence time between ancient humans and modern humans, ancient humans carry many alleles that are specific to their lineage. Consequently, introgression from Neanderthals or Denisovans in modern human genomes was successfully detected by D‐statistics or S* or S*‐like approaches. However, the introgression that we focused on in this work occurred between commercial broiler and CICs. Although commercial chickens experienced strong continued artificial selection, the difference between commercial broiler and CICs was not sufficiently large. The haplotype similarity‐based method would be more naturally suitable, especially in our case, in which the direction of introgression is clear.

Indigenous chicken genetic resources have recently gained increasing attention from researchers. For example, Mekchay and colleagues investigated the population structure of 400 Thai indigenous chickens based on SNPs (Mekchay et al., 2014). A recent study on microsatellites detected potential introgression signature from White Plymouth Rock into Huiyang Bearded chickens (Huang et al., 2016). Here, for the first time, we systematically evaluated gene introgression in Chinese indigenous breeds from commercial broiler. By investigating haplotype similarity at the population level, we detected extensive introgression from WPR to various CICs. Our results not only recovered existing crossbreeding events (Shiqi Za and Kuaida Silkie), but also provided evidence of introgressions from WPR to other CICs. Since the samples that we used in this study were mostly collected from the National Chicken Genetic Resources Center, the introgression had existed extensively before the problem was identified, at least for the chickens we sampled. Interestingly, Tibetan chicken is the least introgressed breed, suggesting that inclement geographical or economic conditions are “protective” for external introgressions. In our study, introgression is accompanied by positive selection. Positive selection facilitates to elevate the frequency of introgression haplotype. Positive selection analyses rejected the neutral model.

Finally, although we detected extensive introgression in CICs, precisely how the introgression occurred remains unclear. Introgression pulses may exist from WPR to CICs independently or indirect introgression may occur through geographically close breeds (Supporting Information Figure S47). For example, the ratio of candidate introgression region shared by SK with KD was as high as 84%, indicating that backcross might be the reason for introgression in SK (Supporting Information Table S9). However, further investigation of this issue is requisite. In addition, other commercial broilers, such as Anak, Ross and Cobb broiler, which were not sampled in this study, were also documented as being introduced to China in the past decades. Introgressions reported here might constitute a mixture, with contributions of possible introgressions from these breeds. It is thus reasonable to regard the introgressions from WPR reported in this study as a representative of all commercial broilers.

## DATA ARCHIVING STATEMENT

Data available from the Dryad Digital Repository: https://doi.org/10.5061/dryad.5sb71.

## CONFLICT OF INTEREST

None declared.
