# Supplementary material for: Widespread introgression in Chinese indigenous chicken breeds from commercial broiler
Source: Evol Appl. 2019 Jan 22;12(3):610–21. doi: 10.1111/eva.12742 (PMC6383742; doi:10.1111/eva.12742)
Supplement: Supplementary file 1 [file EVA-12-610-s001.docx]

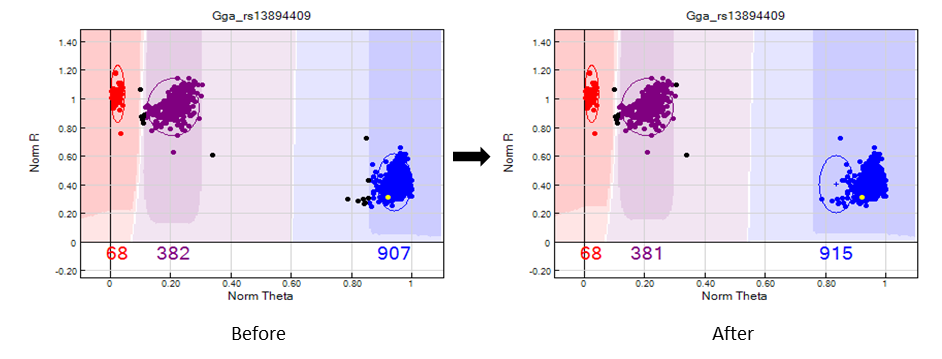


**Figure S1.** Examples of SNP quality editing. Nom R is the normalized allelic intensities; Norm Theta is the normalized the allelic intensity ratio. Genotypes on the right side were manually edited.


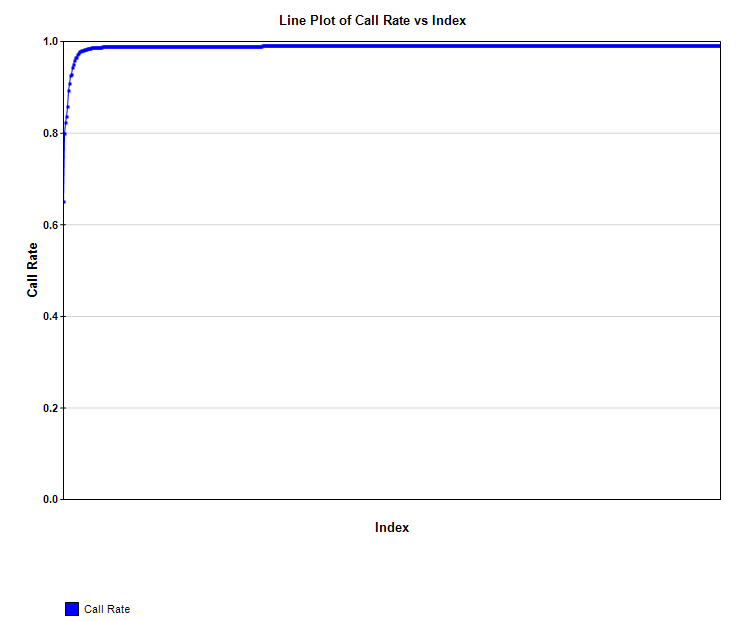


**Figure S2.** Call rate for genotyping samples, including two wild breeds, 10 Chinese indigenous chicken breeds, and White Plymouth Rock.


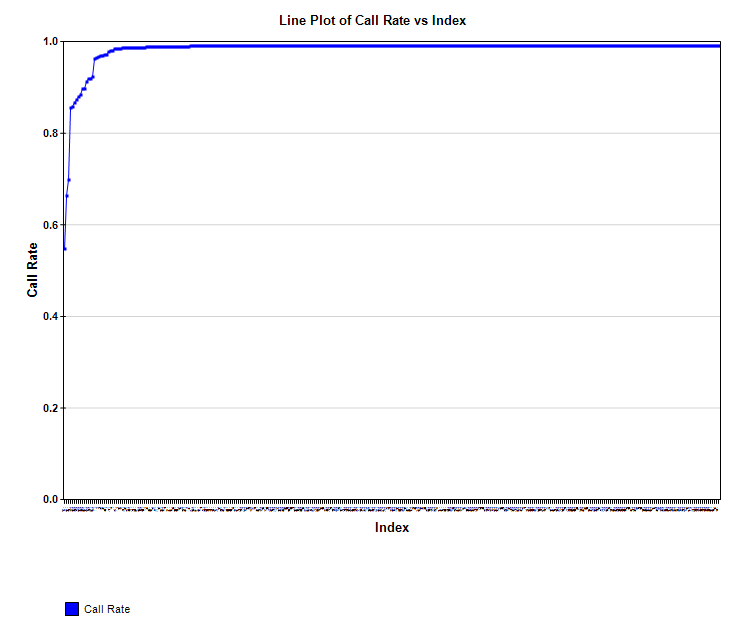


**Figure S3.** Call rate for White Leghorn samples.


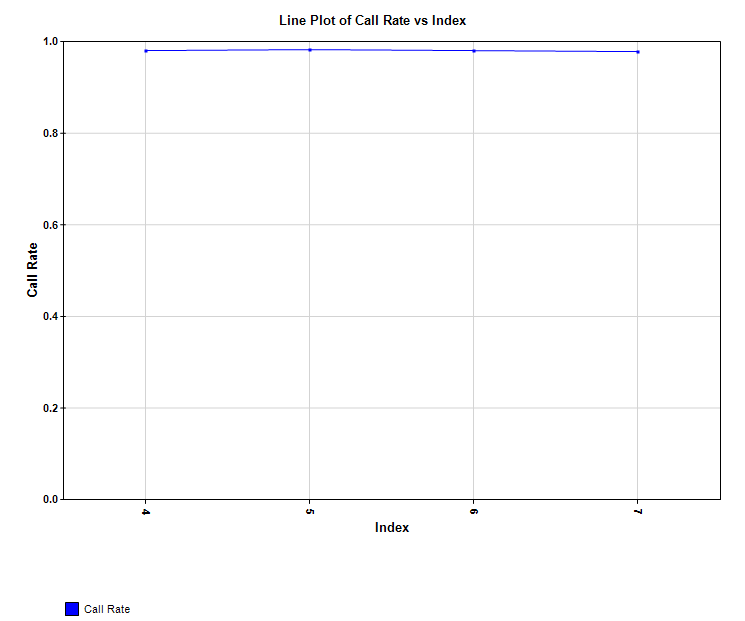


**Figure S4.** Call rate for red jungle fowl samples.


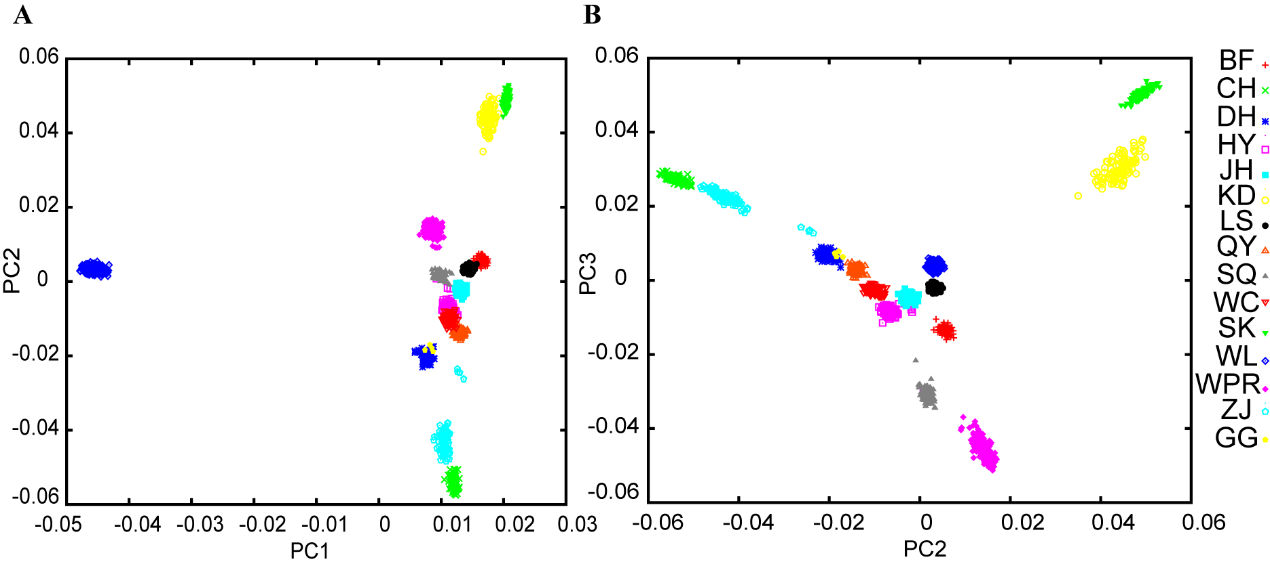


**Figure S5.** Principal component analysis (PCA) for the GENOMESTUDIO output.

| CH | 0.199 |  |  |  |  |  |  |  |  |  |  |  |  |
| --- | --- | --- | --- | --- | --- | --- | --- | --- | --- | --- | --- | --- | --- |
| DH | 0.166 | 0.116 |  |  |  |  |  |  |  |  |  |  |  |
| HY | 0.147 | 0.134 | 0.074 |  |  |  |  |  |  |  |  |  |  |
| JH | 0.164 | 0.154 | 0.103 | 0.096 |  |  |  |  |  |  |  |  |  |
| KD | 0.177 | 0.174 | 0.129 | 0.118 | 0.130 |  |  |  |  |  |  |  |  |
| LS | 0.188 | 0.191 | 0.158 | 0.141 | 0.153 | 0.168 |  |  |  |  |  |  |  |
| QY | 0.156 | 0.130 | 0.076 | 0.078 | 0.104 | 0.126 | 0.149 |  |  |  |  |  |  |
| SQ | 0.169 | 0.166 | 0.117 | 0.095 | 0.126 | 0.145 | 0.166 | 0.117 |  |  |  |  |  |
| WC | 0.143 | 0.122 | 0.064 | 0.061 | 0.091 | 0.114 | 0.136 | 0.070 | 0.100 |  |  |  |  |
| SK | 0.199 | 0.196 | 0.165 | 0.150 | 0.161 | 0.060 | 0.191 | 0.153 | 0.181 | 0.144 |  |  |  |
| WL | 0.290 | 0.260 | 0.214 | 0.218 | 0.242 | 0.265 | 0.276 | 0.232 | 0.232 | 0.213 | 0.299 |  |  |
| WPR | 0.158 | 0.162 | 0.116 | 0.097 | 0.117 | 0.127 | 0.155 | 0.117 | 0.095 | 0.098 | 0.171 | 0.210 |  |
| ZJ | 0.180 | 0.077 | 0.092 | 0.109 | 0.130 | 0.151 | 0.170 | 0.111 | 0.145 | 0.101 | 0.178 | 0.239 | 0.141 |
|  | BF | CH | DH | HY | JH | KD | LS | QY | SQ | WC | SK | WL | WPR |

**Figure S6.** Pair-wise Fst values among populations, except red jungle fowl.


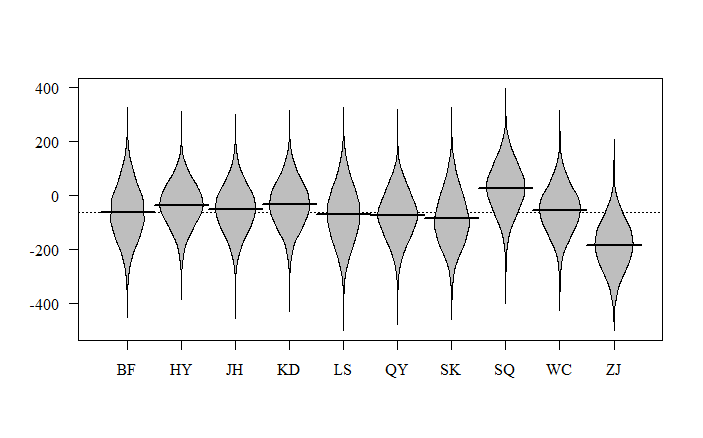


**Figure S7.** Distribution of $\Delta\chi^{2}$ for CICs with the window size equal to five SNPs.


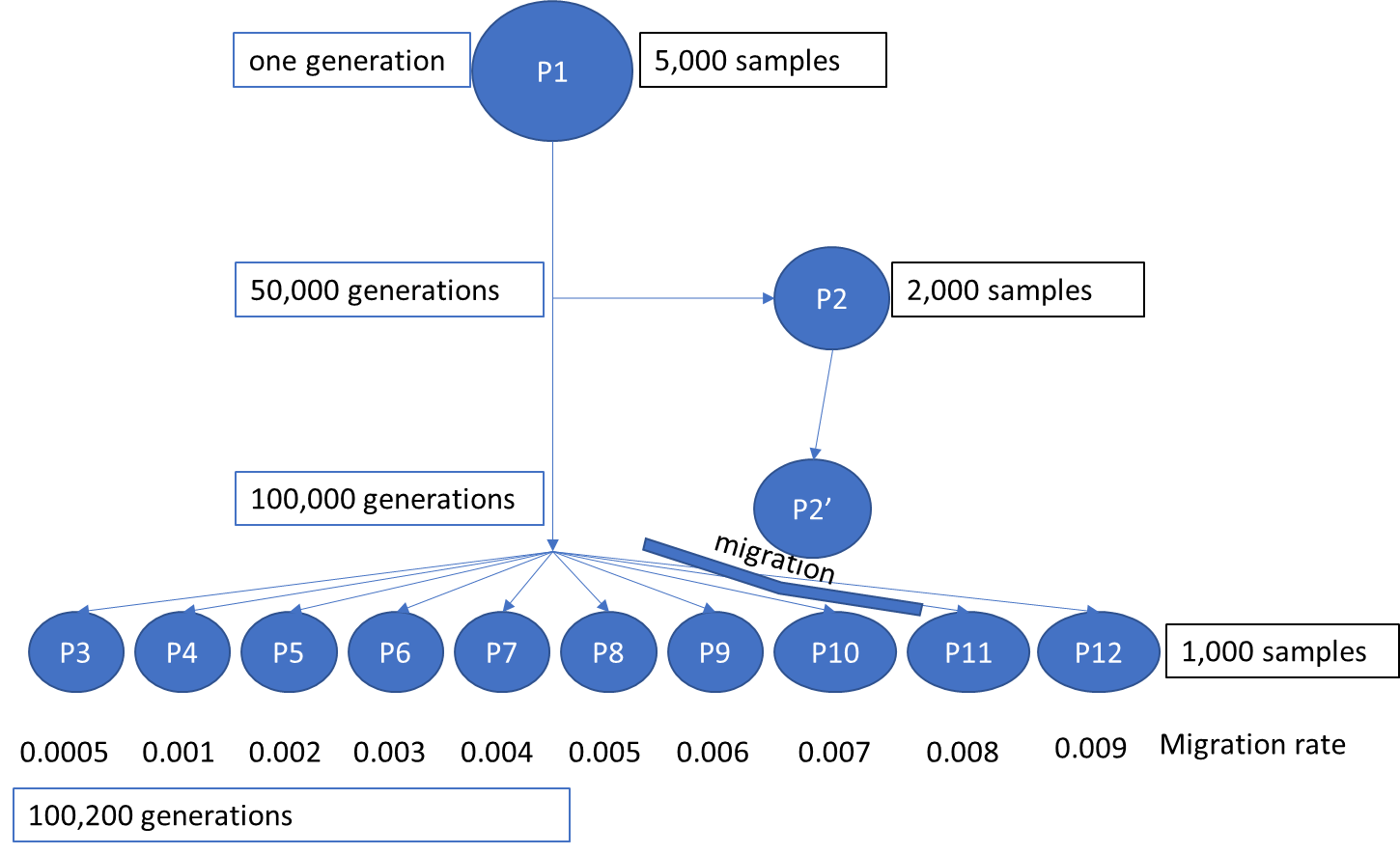


**Figure S8.** Diagram of the simulation model.


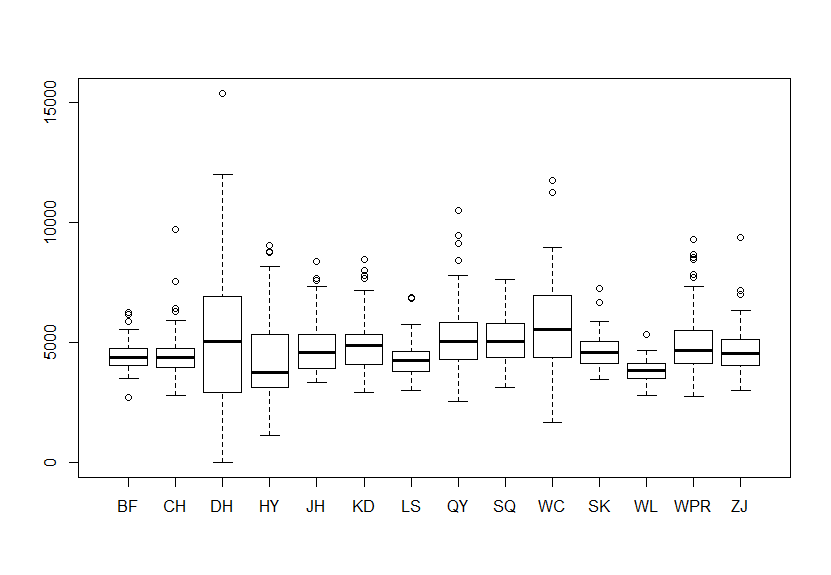


**Figure S9.** The distribution of average ROH lengths for each chicken population.


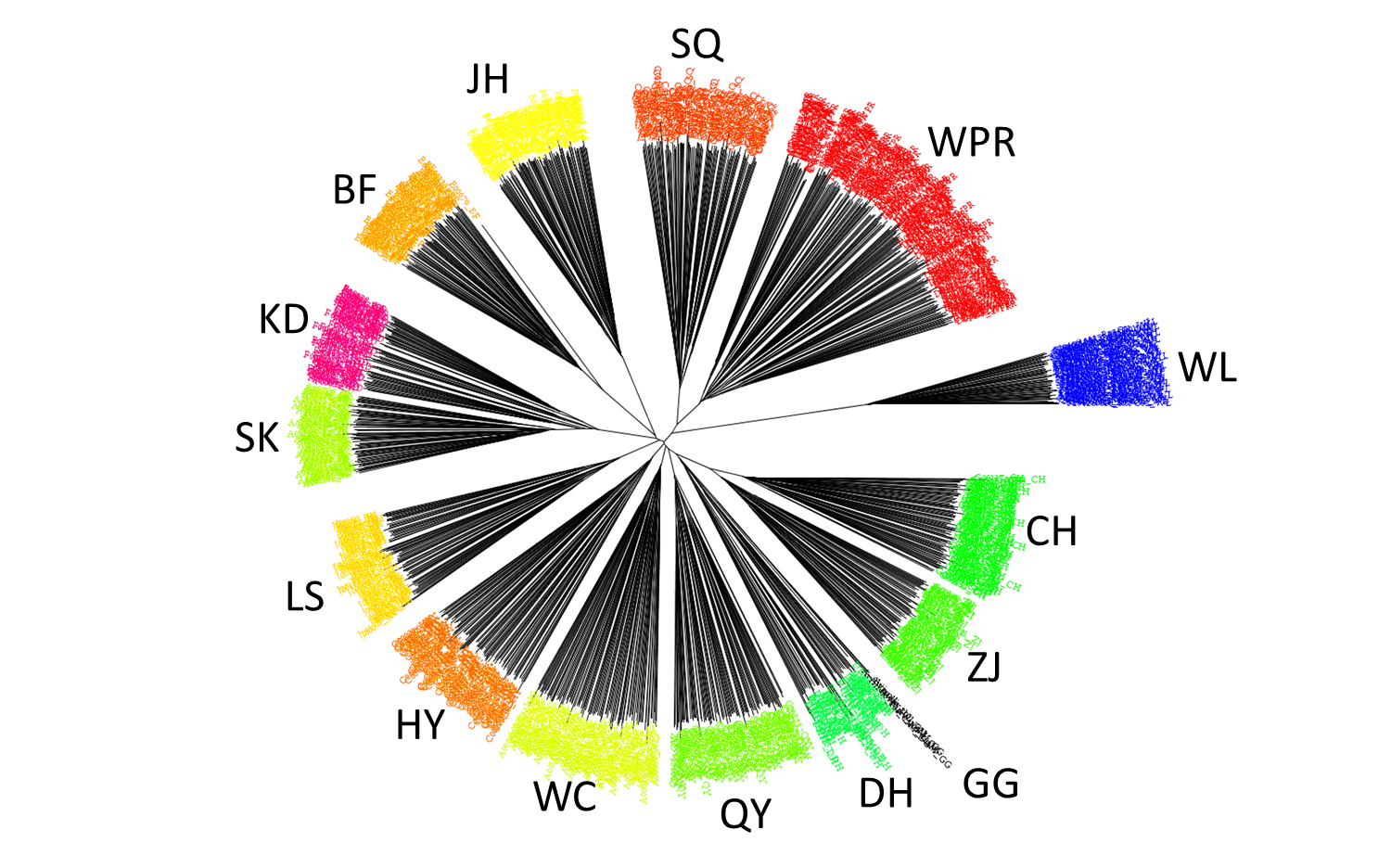


**Figure S10.** IBS tree for all samples.

**Figure S11.** CV error for ADMIXTURE analysis.


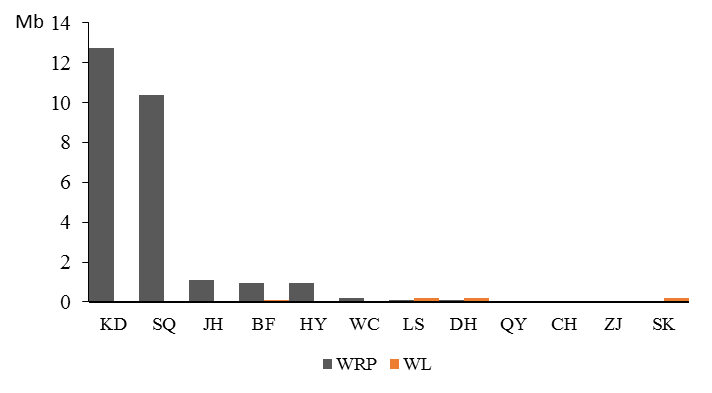


**Figure S12.** Average IBD sharing between CICs and commercial chickens.


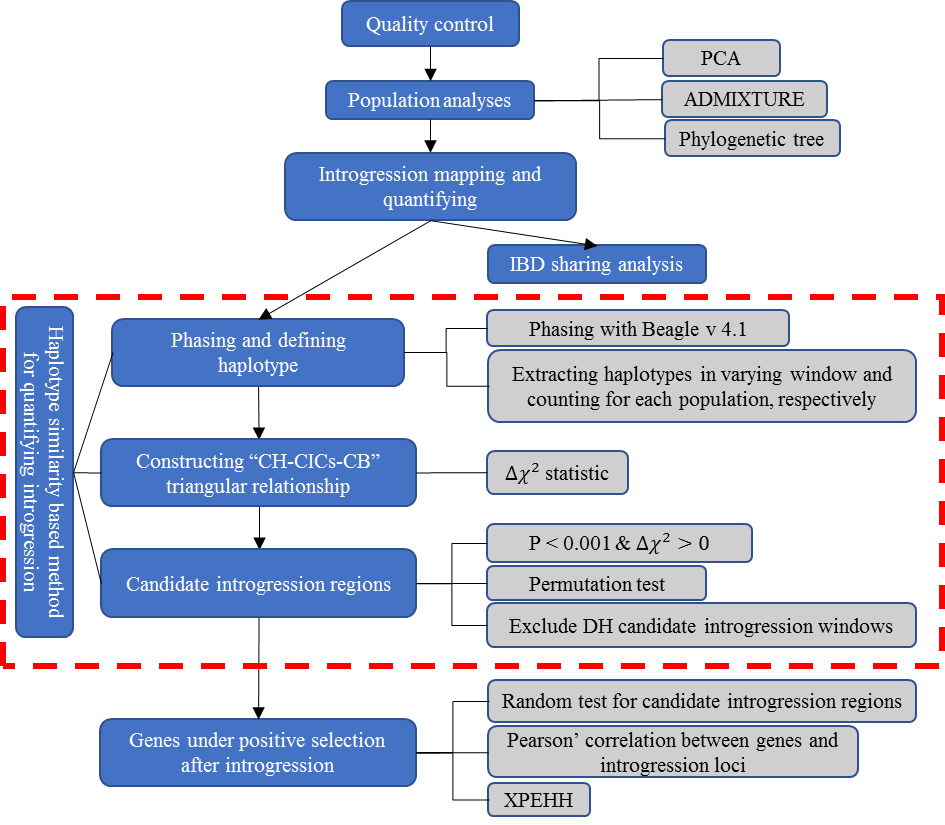


**Figure S13.** Overview of the work flow. The contents in the red dashed box represent the approaches for detecting introgression.


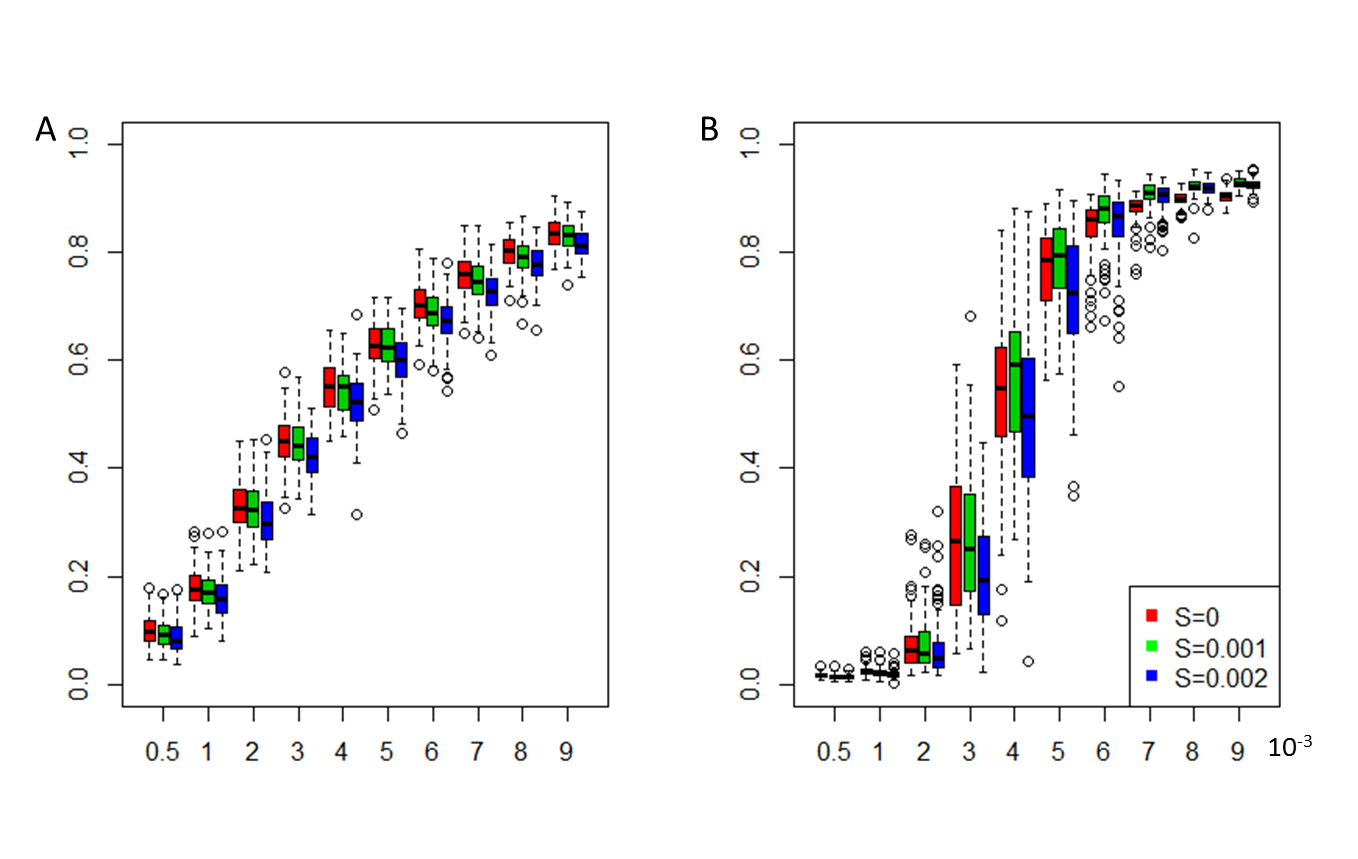


**Figure S14.** Comparison of introgression ratio detection between simulation data and haplotype similarity, based on the Chi-square method. Three scenarios were modeled with positive selection coefficients at s=0 (neutral), s=0.001, or s=0.002. (A) Allele frequency weighted introgression ratio by simulation. (B) Haplotype similarity weighted ratio.

**Figure S15.** Scatter plot for introgression ratio between haplotype similarity, based on the Chi-square method and simulated data (Pearson’s correlation coefficient was 0.958, with p-value < $2.2\times{10}^{-16}$).


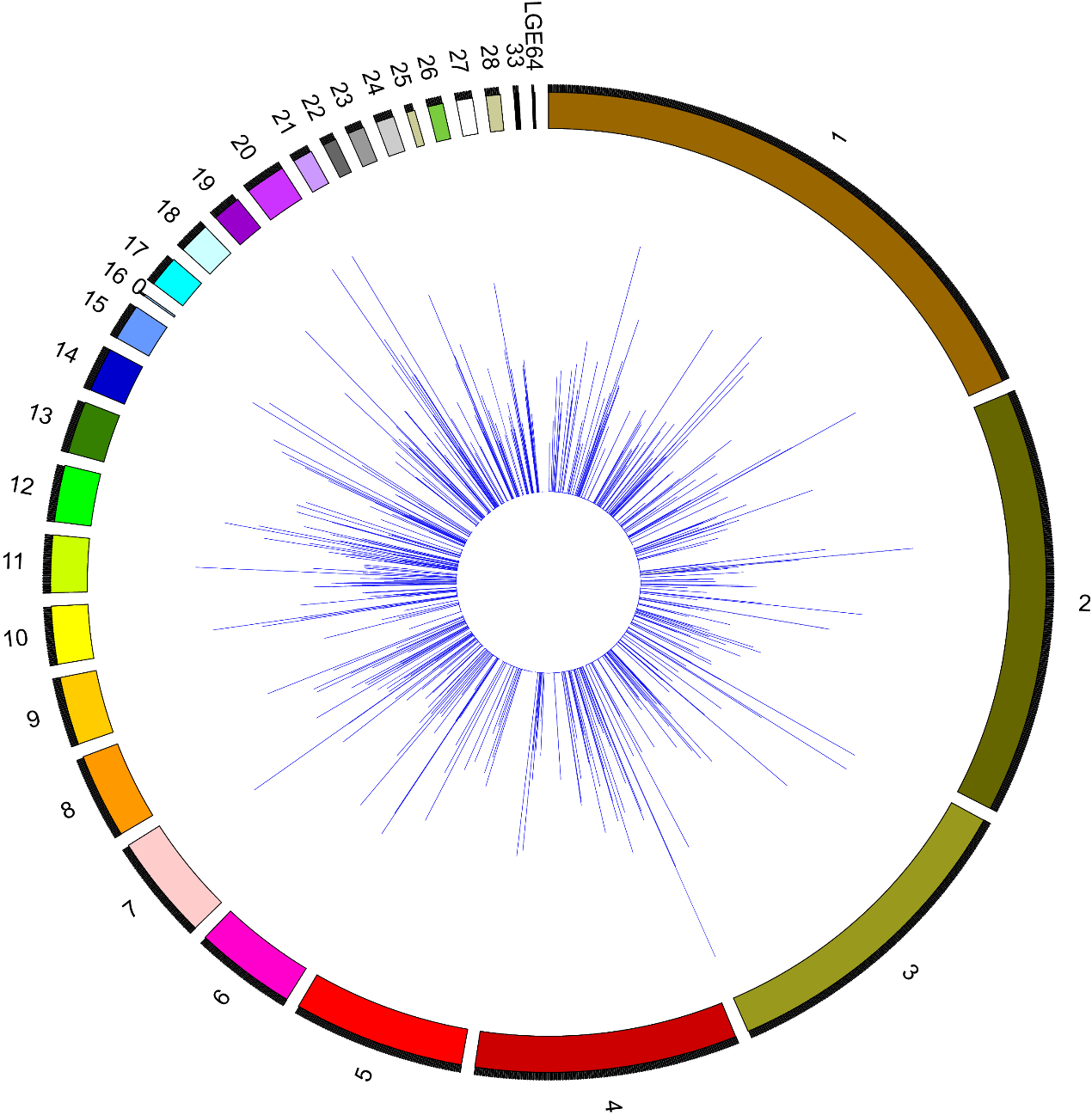


**Figure S16.** Common ancestor for Chinese indigenous chickens that were detected in Dehong. The genome of Dehong was scanned according to the criteria that we developed to detect introgression from WPR. Because red jungle fowl is the ancestor of Chinese indigenous chickens, we regard all of those regions detected in Dehong as false-positives. Candidate introgression regions that overlaped with false-positives will be excluded.


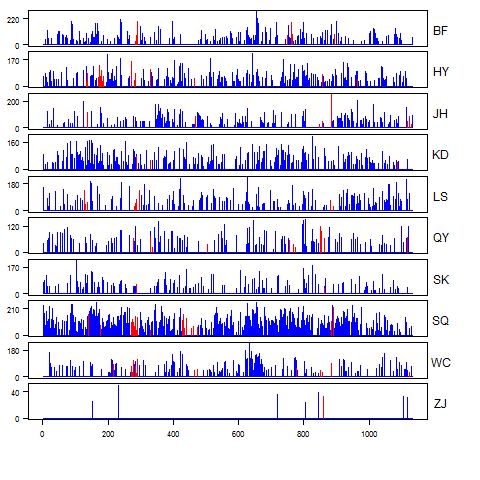


**Figure S17. Physical map and extent of introgression/positive selection on chromosome 2.**


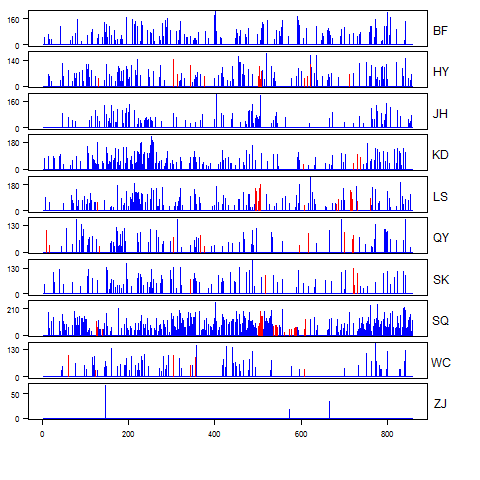


**Figure S18. Physical map and extent of introgression/positive selection on chromosome 3.**

**
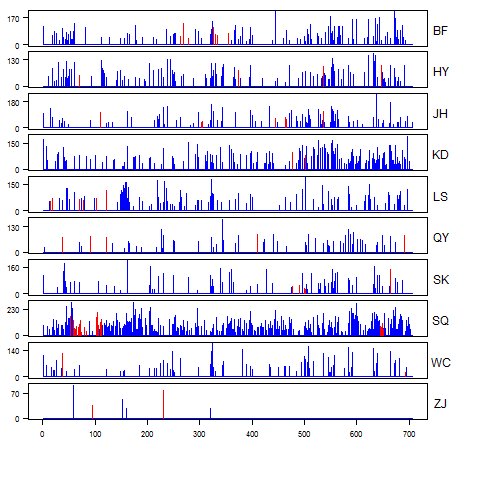
**

**Figure S19. Physical map and extent of introgression/positive selection on chromosome 4.**

**
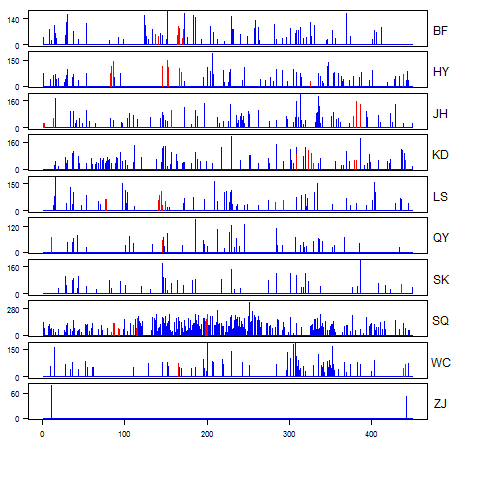
**

**Figure S20. Physical map and extent of introgression/positive selection on chromosome 5.**


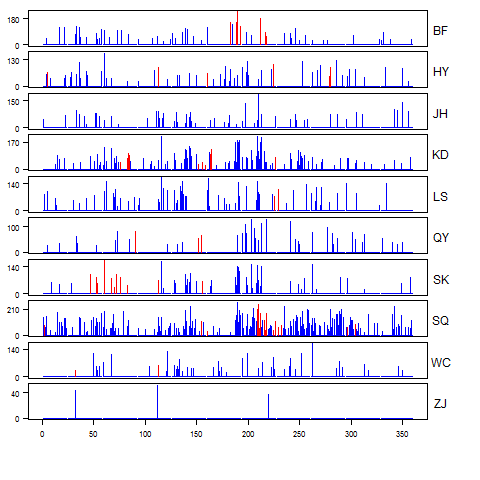


**Figure S21. Physical map and extent of introgression/positive selection on chromosome 6.**


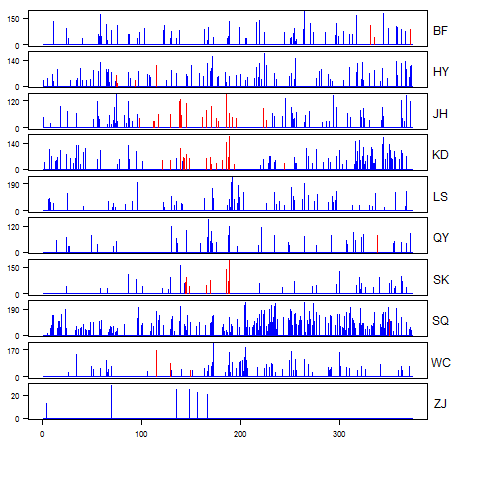


**Figure S22. Physical map and extent of introgression/positive selection on chromosome 7.**


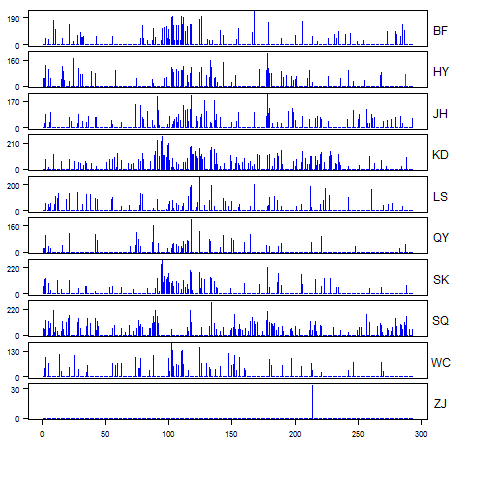


**Figure S23. Physical map and extent of introgression/positive selection on chromosome 8.**


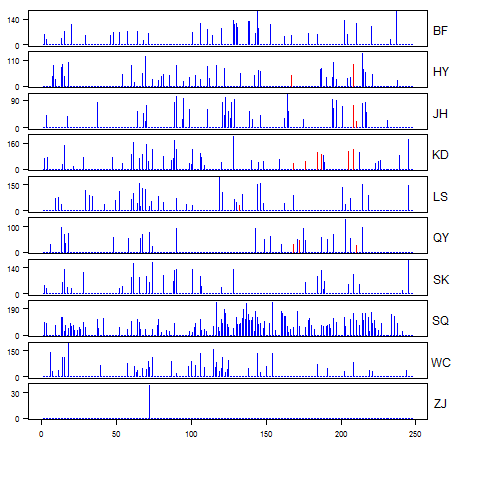


**Figure S24. Physical map and extent of introgression/positive selection on chromosome 9.**


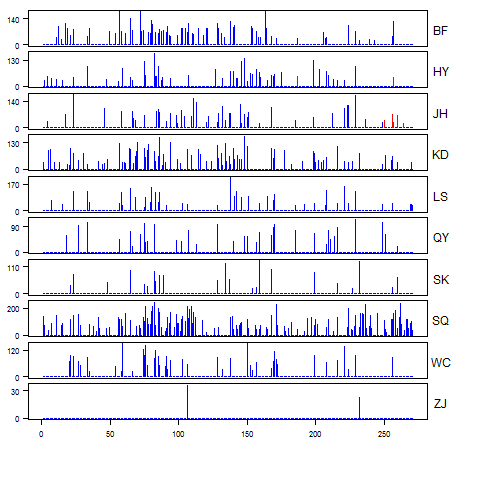


**Figure S25. Physical map and extent of introgression/positive selection on chromosome 10.**


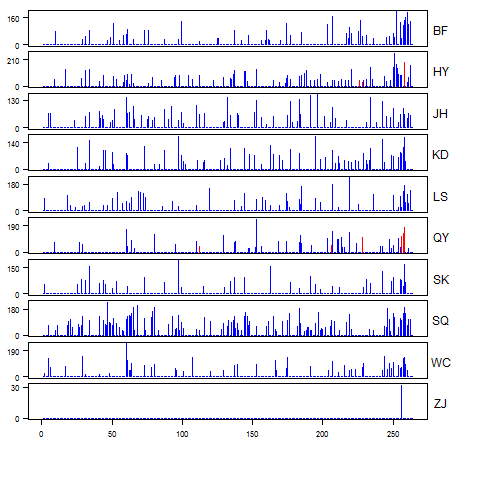


**Figure S26. Physical map and extent of introgression/positive selection on chromosome 11.**


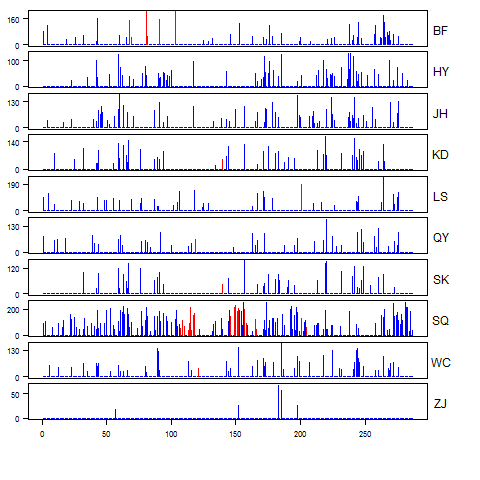


**Figure S27. Physical map and extent of introgression/positive selection on chromosome 12.**


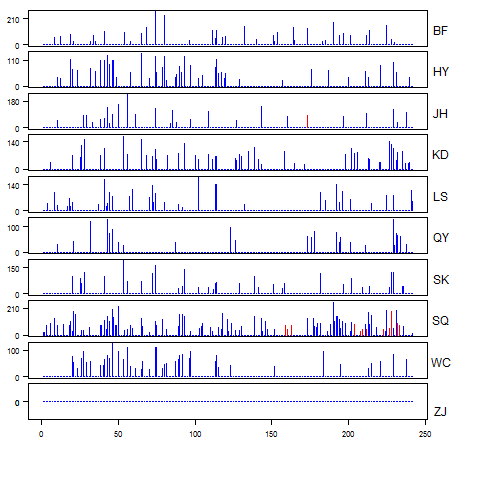


**Figure S28. Physical map and extent of introgression/positive selection on chromosome 13.**


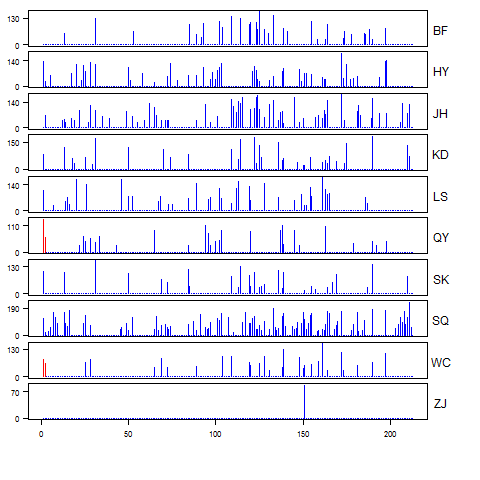


**Figure S29. Physical map and extent of introgression/positive selection on chromosome 14.**


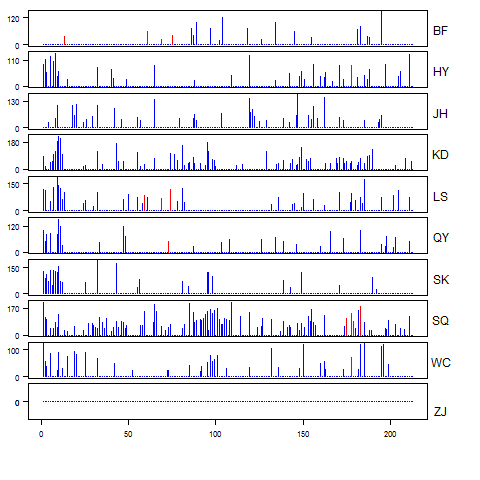


**Figure S30. Physical map and extent of introgression/positive selection on chromosome 15.**


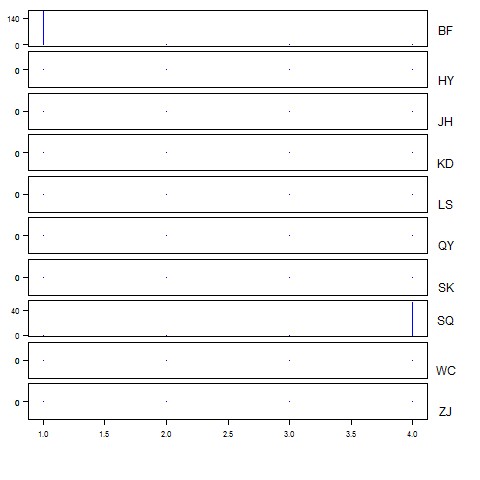


**Figure S31. Physical map and extent of introgression/positive selection on chromosome 16.**


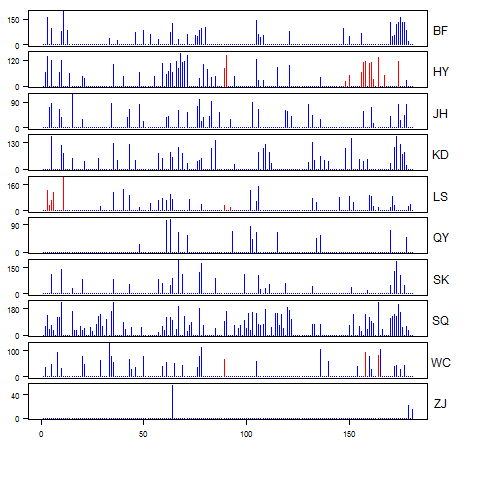


**Figure S32. Physical map and extent of introgression/positive selection on chromosome 17.**


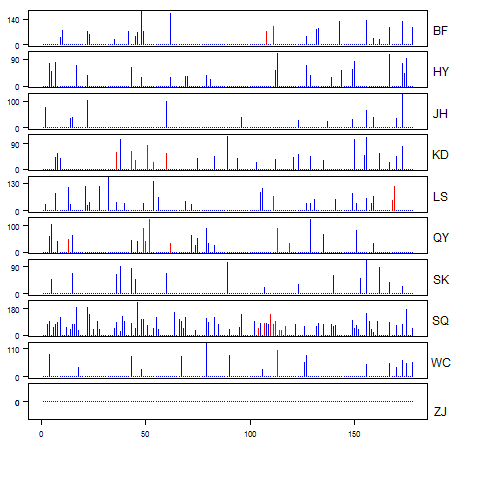


**Figure S33. Physical map and extent of introgression/positive selection on chromosome 18.**


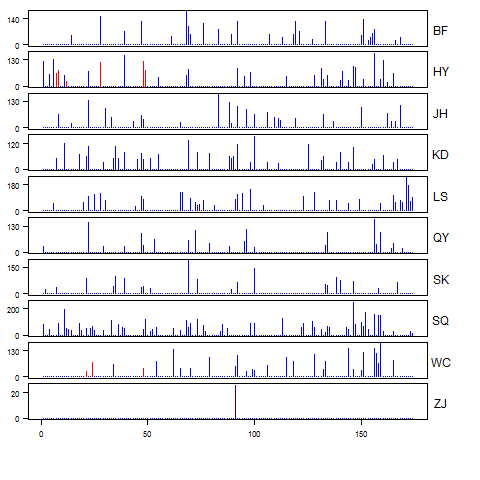


**Figure S34. Physical map and extent of introgression/positive selection on chromosome 19.**


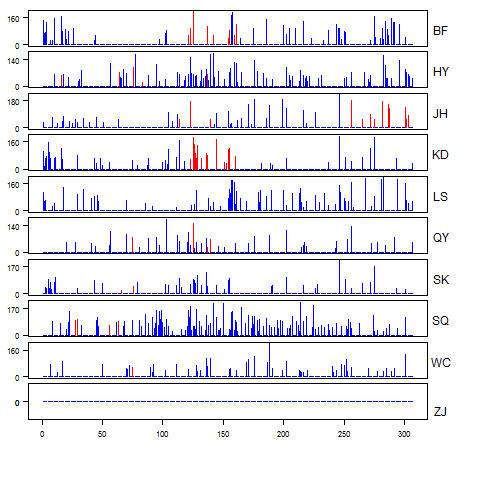


**Figure S35. Physical map and extent of introgression/positive selection on chromosome 20.**


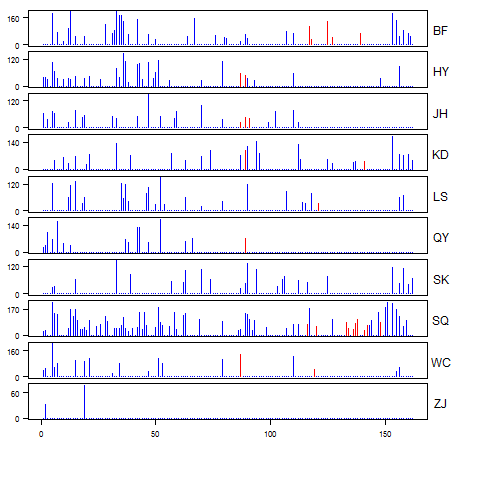


**Figure S36. Physical map and extent of introgression/positive selection on chromosome 21.**


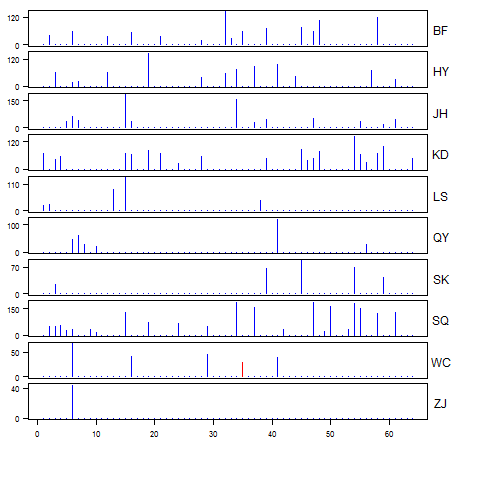


**Figure S37. Physical map and extent of introgression/positive selection on chromosome 22.**


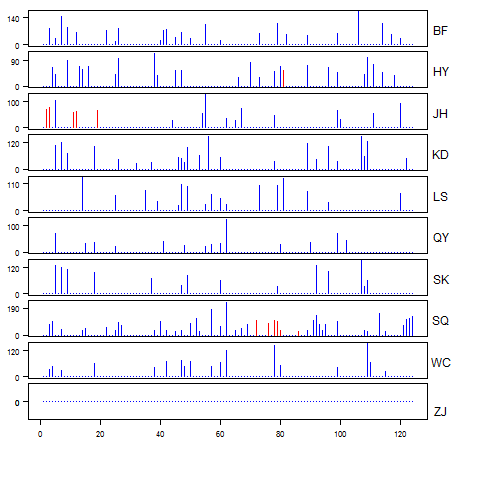


**Figure S38. Physical map and extent of introgression/positive selection on chromosome 23.**


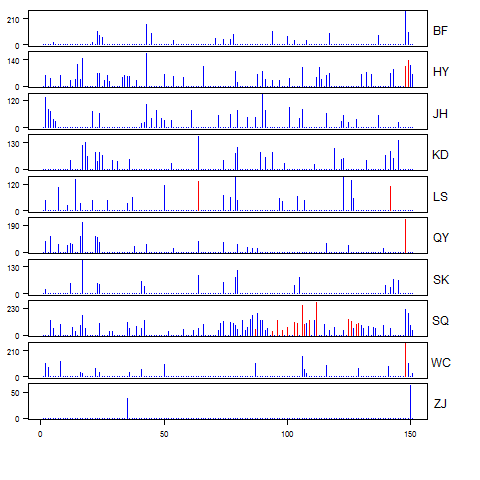


**Figure S39. Physical map and extent of introgression/positive selection on chromosome 24.**


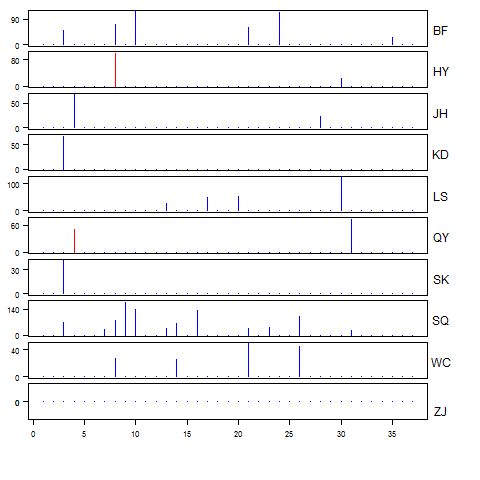


**Figure S40. Physical map and extent of introgression/positive selection on chromosome 25.**


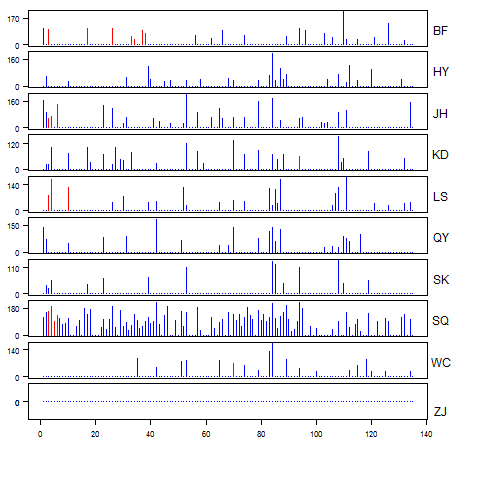


**Figure S41. Physical map and extent of introgression/positive selection on chromosome 26.**


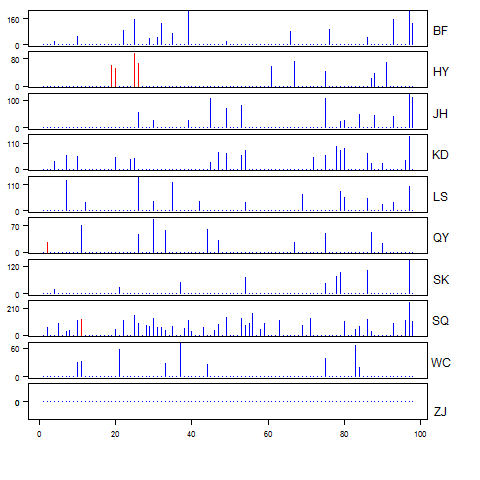


**Figure S42. Physical map and extent of introgression/positive selection on chromosome 27.**


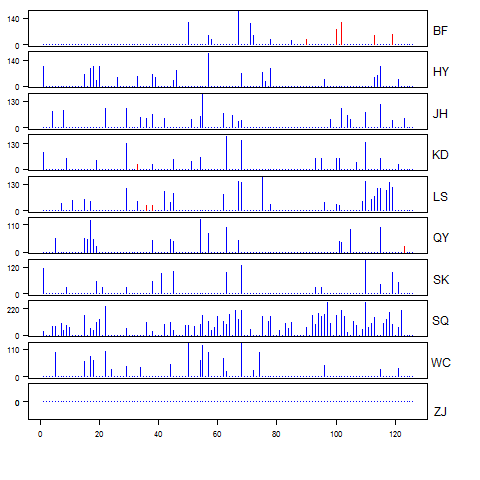


**Figure S43. Physical map and extent of introgression/positive selection on chromosome 28.**


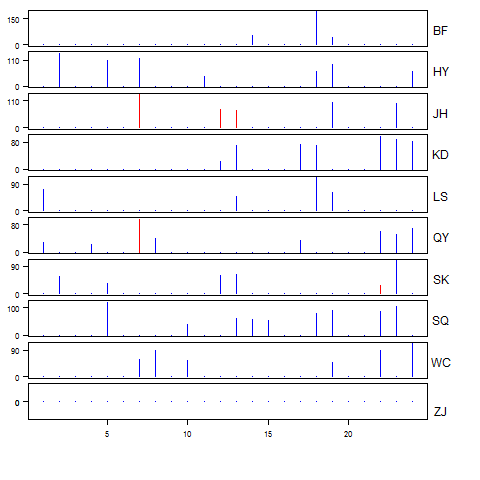


**Figure S44. Physical map and extent of introgression/positive selection on chromosome 33.**


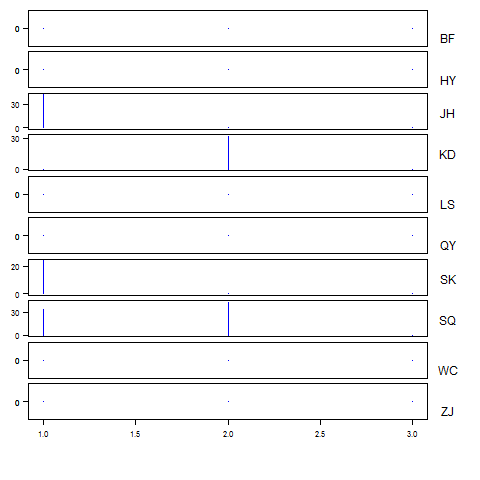


**Figure S45. Physical map and extent of introgression/positive selection on LGE64.**


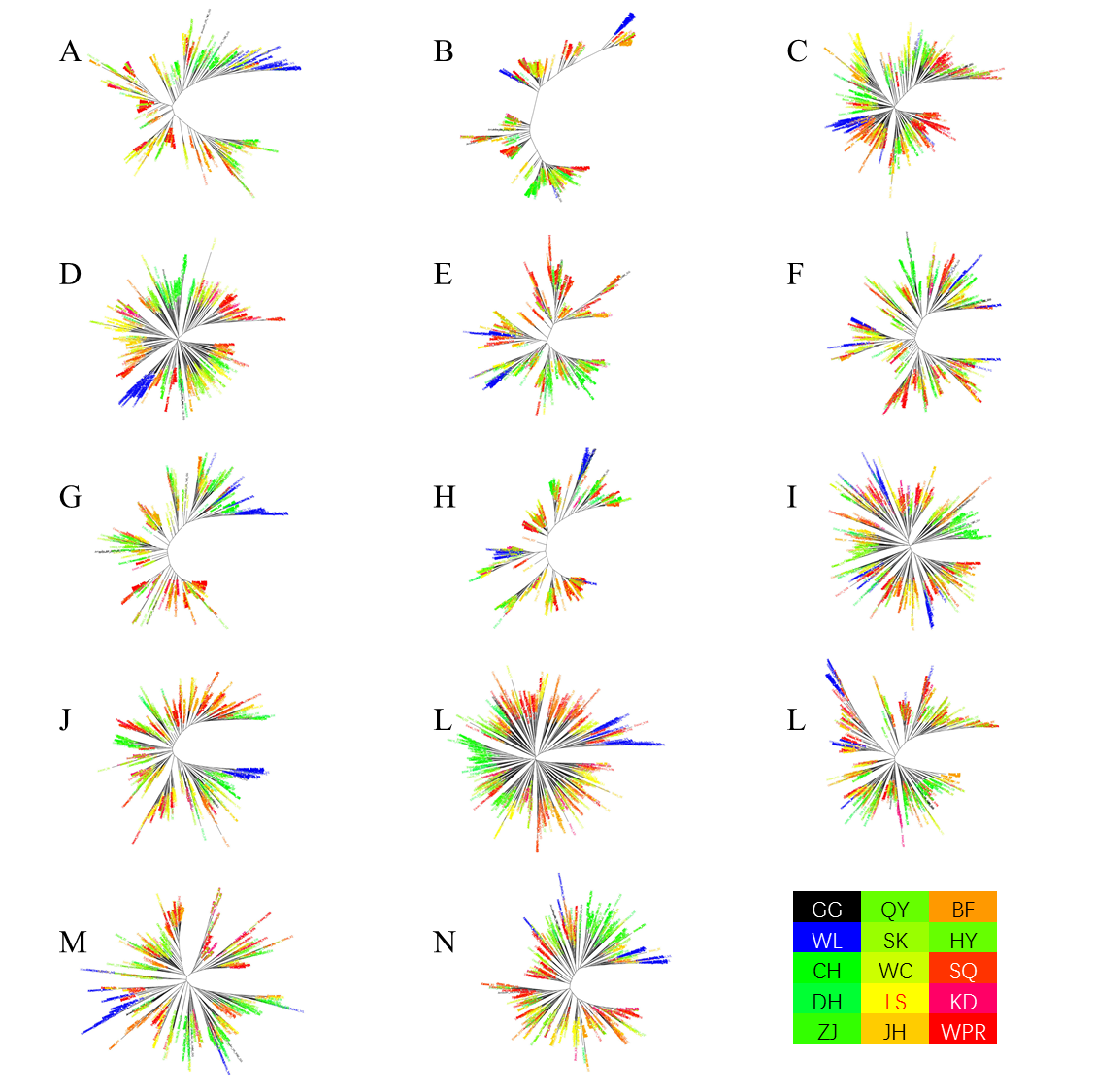


**Figure S46.** IBS tree for all samples of candidate multi-introgression regions. Positions correspond to Table S7, and colors correspond to Figure S10.


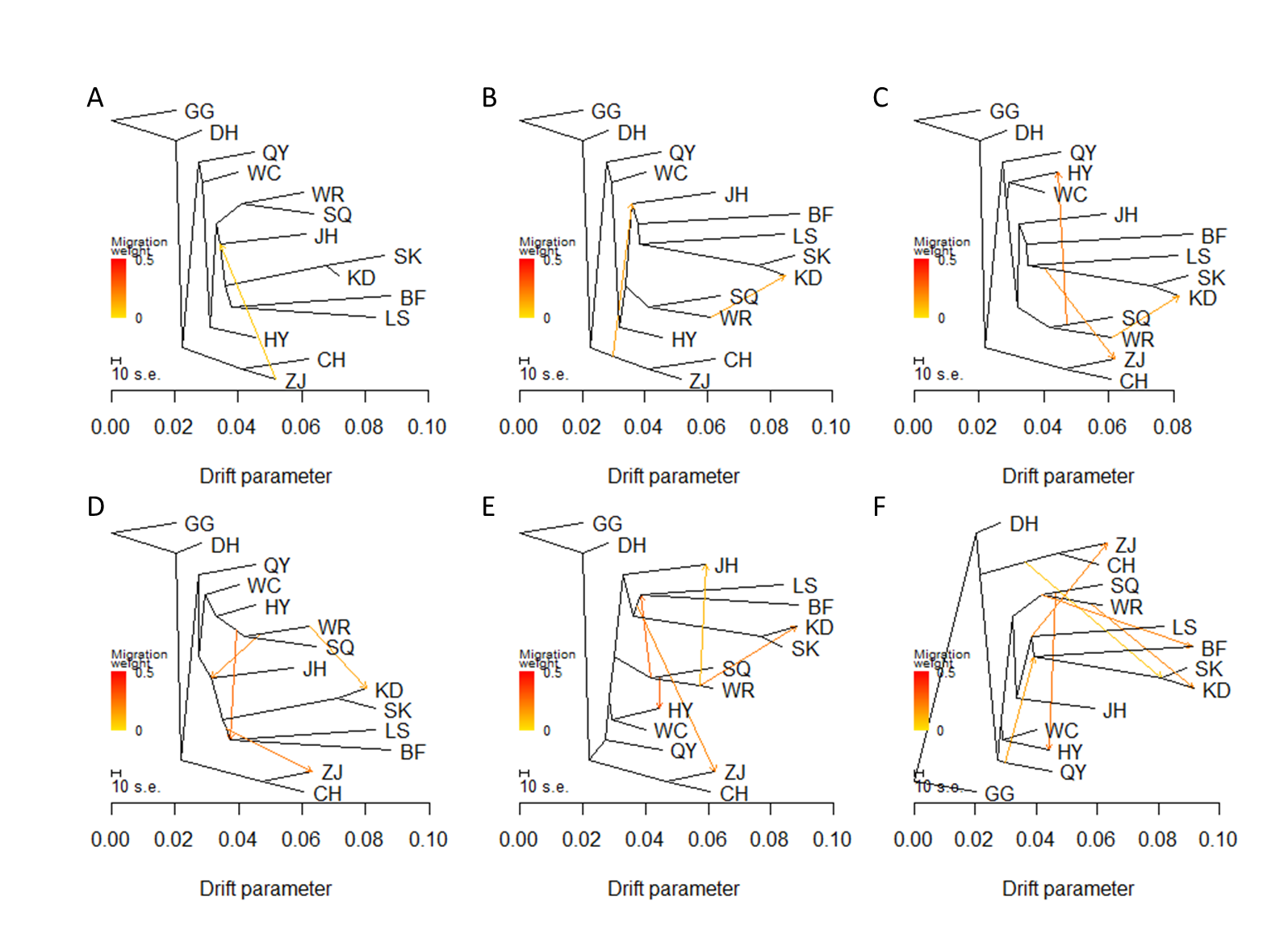


**Figure S47.** TreeMix analyses among populations. The migration that we allowed, varying from one to six corresponding to A to F, respectively.
